# New Models for Estimating the Sorption of Sulfonamide and Tetracycline Antibiotics in Soils

**DOI:** 10.3390/ijerph192416771

**Published:** 2022-12-14

**Authors:** Jinsheng Hu, Xiangyu Tang, Minghui Qi, Jianhua Cheng

**Affiliations:** 1 Institute of Mountain Hazards and Environment, Chinese Academy of Sciences, Chengdu 610041, China; 2 University of Chinese Academy of Sciences, Beijing 100049, China; 3 State Key Laboratory of Subtropical Silviculture, Zhejiang A&F University, Hangzhou 311300, China

**Keywords:** affinity coefficient, governing factor, pedotransfer function, hydrophobic interactions, electrostatic interactions, hydrogen bonding

## Abstract

Sulfonamides (SAs) and tetracyclines (TCs) are two classes of widely used antibiotics. There is a lack of easy models for estimating the parameters of antibiotic sorption in soils. In this work, a dataset of affinity coefficients (*K*_f_ and *K*_d_) of seven SA/TC antibiotics (i.e., sulfachlorpyridazine, sulfamethazine, sulfadiazine, sulfamethoxazole, oxytetracycline, tetracycline, and chlortetracycline) and associated soil properties was generated. Correlation analysis of these data showed that the affinity coefficients of the SAs were predominantly affected by soil organic matter and cation exchange capacity, while those of the TCs were largely affected by soil organic matter and pH. Pedotransfer functions for estimating *K*_f_ and *K*_d_ were built by multiple linear regression analysis and were satisfactorily validated. Their performances would be better for soils having higher organic matter content and lower pH. These pedotransfer functions can be used to aid environmental risk assessment, prioritization of antibiotics and identification of vulnerable soils.

## 1. Introduction

In recent years, antibiotics have often been detected in soils at elevated levels as a result of their discharge after human and animal use [1,2]. Soil pollution with antibiotics has been recognized as a potential threat, potentially causing the development and spread of antibiotic resistance in soil microbes, impairment of soil ecosystem functions, contamination of agricultural products, and offsite pollution of receiving water bodies via hydrological processes [3,4].

Sorption regulates the distribution of antibiotics between aqueous and solid phase in soils, thus affecting their mobility, bioavailability, and fate [5,6,7]. For instance, sulfonamides (SAs) are the most mobile antibiotics in soils due to their low sorption coefficient, and their residues in soils range from ng kg^−1^ to μg kg^−1^ level [8]. Contrastingly, tetracyclines (TCs), as another class of antibiotics commonly used, are less mobile and show higher residues in soils ranging from 12 to 100 μg kg^−1^ [9]. Sorption behavior is dependent on antibiotic physico-chemical properties (e.g., octanol–water partitioning coefficient (*K*_ow_), acid dissociation constant (p*K*_a_), and molecular structure) and soil properties (e.g., organic matter content, pH, cation exchange capacity, and texture) [10,11]. Parameters of sorption isotherm models, such as affinity coefficient (*K*_f_) and linearity coefficient (*n*) of the Freundlich model and affinity/distribution coefficient (*K*_d_) of the linear model, are key components in environmental risk assessment of antibiotics [12]. In an area where a large number of antibiotics are in use, it may be infeasible to experimentally determinate sorption parameters of all antibiotics in all soils of concern, due to the budget and time limitations. There is clearly a need to develop models for estimating sorption parameters.

Antibiotics are mostly polar, ionizable compounds. In soil’s aqueous phase, ionizable antibiotics (e.g., sulfonamides, tetracyclines, and fluoroquinolones) can exist as cation, anionic, neutral, or zwitterion species, depending on their p*K*_a_ and solution pH [12,13,14]. A number of mechanisms, such as hydrophobic interactions with soil organic matter (SOM), hydrogen and covalent bonding to SOM, exchange of cationic antibiotic species with cations on negatively charged surfaces of SOM and phyllosilicate clay minerals, surface complexation of anionic antibiotic species on surficial Fe/Mn oxides and clay mineral edge sites, cation bridging of anionic antibiotic species to negatively charged sites on clay minerals and organic matter, and electrostatic attraction of anionic antibiotic species with positively charged Fe/Al oxides, can be involved in the sorption of antibiotics [11,12,15,16,17,18,19,20,21,22]. For a given antibiotic, it is reasonable to consider parameters concerning antibiotic speciation, soil components, and environmental conditions as potential inputs of models for estimating sorption parameters. Differences in model formulation among antibiotics can reflect differences in main sorption mechanisms and their relative contributions.

Models for estimating sorption parameters can be developed using different approaches. Statistical regression analyses are traditional approaches to establishing linear or nonlinear quantitative models relating to the sorption parameters of antibiotics with soil properties [23,24]. Regression-based models using antibiotic physico-chemical properties alone as inputs were also developed, and the performance of such models can be improved by also including soil properties as inputs [25,26,27]. Moreover, satisfactory estimation of sorption parameters was obtained using machine learning approaches (e.g., artificial neural network, and random forest), which can involve many more inputs than regression-based approaches can do [28,29]. It should be noted that some soil properties (e.g., exchangeable K, Na, and Mg) used as inputs of machine learning models are not commonly reported in the literature, potentially limiting their broader applications. Overall, regression-based models are recognized as the most practical tools for estimating antibiotic sorption parameters as they are explicitly programmed and require only readily available inputs. Nevertheless, such models developed in each of most previous studies were based only on sorption data for soils of a single country or geological region [2,6,10,13,24,26,30,31,32,33], and their application in soils of other countries/regions may be problematic due to their inherent site-specific nature.

The aim of this study was therefore to develop new regression-based models for estimating sorption parameters of seven widely used SA/TC antibiotics, which were expected to be applicable in soils of different countries/regions. The specific objectives of this study were to (a) establish a dataset of sorption parameters for the target antibiotics in a wide range of soil properties based on data from the literature; (b) identify key factors affecting antibiotic sorption in soils and underlying mechanisms; and (c) develop and validate new models for estimating the sorption parameters of the target antibiotics which can be used in combination with spatial information on soil properties to evaluate the environmental risk of antibiotics on a global scale.

## 2. Materials and Methods

### 2.1. Physical and Chemical Properties of Antibiotics

Seven antibiotics, including four SAs (sulfachlorpyridazine (SCP), sulfamethazine (SMT), sulfadiazine (SDZ), and sulfamethoxazole (SMX)) and three TCs (oxytetracycline (OTC), tetracycline (TC), and chlortetracycline (CTC)), were selected for this study. Their physiochemical properties are provided in Table 1. The proportion of their species in soil water is dependent on their p*K*_a_ and soil pH [34]. All the SAs are hydrophilic (log*K*_ow_ ≤ 0.89), and so are the TCs, except CTC which has the greatest hydrophobicity (log*K*_ow_ = 2.07).

### 2.2. Data Collection

A total of 104 publications, of which 10, 21, 20, 18, 38, 22, and 17 were related to SCP, SMT, SDZ, SMX, OTC, TC, and CTC, respectively, were reviewed to create a dataset consisting of sorption parameters of sulfonamide and tetracycline antibiotics and properties of tested soils. This dataset covers 5 continents (Asia, Europe, South America, North America, and Oceania), 14 countries, 3 temperature zones (southern temperate, northern temperate, and tropical), and 10 climate types. Only sorption studies in natural soils were included. Three sorption parameters (*K*_f_ and *n* of the Freundlich model, and *K*_d_ of the linear model) and six soil properties (pH, organic matter (OM) content, cation exchange capacity (CEC) and soil texture (sand, silt, and clay content)) were selected, and their mean values are shown Tables S2–S8. Soil OM content was converted to soil organic carbon (OC) content using the relationship %OM = 1.724 × (%OC) when needed [39]. A high diversity of texture and properties was represented by the soils in this dataset. Experimental parameters (i.e., initial antibiotic concentration in aqueous phase, solid/liquid ratio), which could affect antibiotic sorption for batch experiments [31,40], were also included. The initial antibiotic concentration and solid/liquid ratio ranged from 0.04 to 14,236.00 mg L^−1^ and from 1:1 to 1:625, respectively. This dataset was divided into four independent sub-datasets (“A” and “a” for *K*_f_, or “B” and “b” for *K*_d_). Sub-datasets “A” and “B” (number of *K*_f_ data: 68, 107, 53, 49, 104, 84, and 73 for SCP, SMT, SDZ, SMX, OTC, TC, and CTC, respectively; number of *K*_d_ data: 80, 114, 83, 57, 94, 67, and 72 for SCP, SMT, SDZ, SMX, OTC, TC, and CTC, respectively) was used to build pedotransfer functions for estimating the affinity coefficients of the seven target antibiotics, and sub-datasets “a” and “b” (number of *K*_f_ data: 15, 35, 24, 10, 29, 23, and 20 for SCP, SMT, SDZ, SMX, OTC, TC, and CTC, respectively; number of *K*_d_ data: 18, 39, 21, 15, 27, 23, and 16 for SCP, SMT, SDZ, SMX, OTC, TC, and CTC, respectively) were used to validate the established models.

### 2.3. Sorption Isotherms

The sorption of SAs and TCs on soil is usually described by the linear or Freundlich models, which can be written as Equations (1) and (2), respectively.

(1) $$Q_{e}=K_{d}C_{e}$$

(2) $$Q_{e}=K_{f}C_{e}^{\frac{1}{n}}$$

where *Q*_e_ (mg kg^−1^) is the amount of antibiotic sorbed on the soil at equilibrium; *C*_e_ (mg L^−1^) is the equilibrium concentration of antibiotic in aqueous phase; *K*_d_ (L kg^−1^) is the linear affinity/distribution coefficient; *K*_f_ (mg^1−1/*n*^ L^1/*n*^ kg^−1^) is the Freundlich affinity coefficient; and *n* is the Freundlich linearity index. When the value of *n* is close to 1, Freundlich models are approximately equal to linear models. *n* > 1 indicates the saturation of sorption sites at high concentrations, which hinders the sorption process. *n* < 1 indicates that the previously sorbed antibiotic increases the sorption power of the soil [41].

The *Q*_e_ can be calculated as follows [6]:

(3) $$Q_{e}=\left(C_{i}-C_{e}\right)\frac{V_{w}}{m_{s}}$$

where *C*_i_ is the initial aqueous antibiotic concentration (mg L^−1^), *V*_w_ is the aqueous volume (mL), and *m*_s_ is the soil mass (g).

For studies in which only the Freundlich model was used, Equations (1)–(3) were used to estimate *K*_d_ by re-fitting to the data of initial concentrations and equilibrium concentrations estimated from the reported *K*_f_ and *n* of the Freundlich model. Only the *K*_d_ values estimated with good fittings (*p* < 0.05) were included in the dataset.

### 2.4. Statistics and Modeling

Using the data shown in Tables S2–S8, Pearson correlations of sorption parameters with soil properties were analyzed to reveal the governing factors and mechanisms of antibiotic sorption in soils. Subsequently, multiple linear regressions were performed using SPSS 22.0 software (IBM Corp., Armonk, NY, USA) to develop pedotransfer functions for estimating the parameters of the linear and Freundlich models. Regression-based modeling using both edaphic and non-edaphic variable(s) as inputs was also conducted.

### 2.5. Model Evaluation

The applicability and accuracy of the pedotransfer functions were assessed using the adjusted determination coefficient (*r*^2^), Nash-Sutcliffe efficiency (NSE), root-mean-square error (RMSE), and absolute error (AE). NSE was calculated using Equation (4) to assess the model predictive capability [23]. RMSE and AE were obtained to measure the average magnitude of error in estimation using Equations (5) and (6), respectively. In addition, the percentage of RMSE over the standard deviation (SD) of the reported/re-fitted model parameters was also calculated.

(4) $$\mathrm{NSE}=1-\left[\frac{\sum_{i=1}^{N}{\left(M_{i}-E_{i}\right)}^{2}}{\sum_{i=1}^{N}{\left(M_{i}-M_{\mathrm{mean}}\right)}^{2}}\right]$$

(5) $$\mathrm{RMSE}=\sqrt{\frac{\sum_{i=1}^{N}{\left(M_{i}-E_{i}\right)}^{2}}{N}}$$

(6) $$\mathrm{AE}=\left|\frac{M_{i}-E_{i}}{M_{i}}\right|$$

where *M_i_* and *E_i_* are the *i*th measured and estimated values, respectively. *M*_mean_ is the average of measured data, and *N* is the number of measurements. NSE, which can range from −∞ to 1, was used to evaluate how well the estimation was. The closer NSE is to 1, the better the model can perform. An RMSE value of 0 indicates a perfect fit.

## 3. Results

### 3.1. Distribution of Soil Properties

With respect to individual antibiotics, basic descriptive statistics of the physicochemical properties of the selected soils in the dataset generated in our study are presented in Table S1. For a specific target antibiotic, the number of soils used was in the range of 79–159. The dataset covers a broad range of soil characteristics, reflecting a large variability in source and nature of soils. Soil pH varied from 2.75 to 9.40, with 75.0% of the soils being in the acidic range. pH distribution of the soils used for each target antibiotic and main antibiotic species in soil water are shown in Table S9. In the soils included in the dataset, both the SAs and the TCs were mainly present in neutral/zwitterionic and/or anionic species, with SMX showing the highest dominance of anion forms. Soil OC content ranged from 0.1% to 21.3%, with the median being below 3.0%. Soil CEC was highly variable between 3.40 and 740.00 mmol kg^−1^, with the median being in the range of 61.00–155.00 mmol kg^−1^. Regarding soil texture, more than 50.0% of the total number of soils belong to the clay loam group according to the international soil classification system (Tables S2–S8).

### 3.2. Distribution of Antibiotic Sorption

Sorption parameters for each antibiotic varied greatly among the soils, especially those for the TCs that exhibited high sorption in the soils (Tables S2–S8). Table 2 shows basic descriptive statistics of the reported sorption parameters. Across the SAs, *K*_f_ and *K*_d_ varied from 0.13 to 16.00 mg^1−1/*n*^ L^1/*n*^ kg^−1^ and from 0.02 to 28.50 L kg^−1^, respectively, and were mainly in the low value ranges (1.65–5.25 mg^1−1/*n*^ L^1/*n*^ kg^−1^ and 2.05–4.42 L kg^−1^, respectively). For the SA antibiotics, the median of *K*_f_ and *K*_d_ followed the order of SCP > SMT > SMX > SDZ and SCP > SMT > SMX ≈ SDZ, respectively. Both mean and median of *n* for the SAs were close to 1 (i.e., linear isotherms), indicating that the distribution between the aqueous and solid phase was independent of the amount of antibiotic addition [30]. Both *K*_f_ and *K*_d_ of the TCs (0.28–8176.99 mg^1−1/*n*^ L^1/*n*^ kg^−1^ and 10.06–4473.20 L kg^−1^, respectively) were, at a maximum, three orders of magnitude greater than those of the SAs, which can be mainly attributed to the greater aromaticity of the TCs. It has been known that the antibiotics of greater aromaticity can be more strongly sorbed by soil organic matter, which is known to be highly aromatic [42,43]. For the TC antibiotics, the median of both *K*_f_ and *K*_d_ followed the same order of CTC > OTC > TC. Both mean and median of *n* for the TCs were about 2 (i.e., nonlinear isotherms), indicating that a decreasing tendency for sorption on heterogeneous soil surfaces with increasing initial TC concentration [30]. Moreover, the Freundlich model would provide a better fit for the sorption isotherms of the TCs than the linear model, which was also found by previous studies [30,44].

### 3.3. Correlations between Antibiotic Sorption and Soil Properties

The large variability of sorption parameters across the very dissimilar soils allows the analysis of their correlations with soil properties. Results of the Pearson correlation analysis are presented in Table 3.

*K*_f_ and *K*_d_ of the SAs were positively correlated with OC and CEC (*p* < 0.05), with an exception of the *K*_f_ of SDZ showing no correlation with CEC, indicating hydrophobic interactions and cation exchange were two main sorption mechanisms. The markedly higher correlation coefficient (*r*) values of OC with *K*_f_/*K*_d_, compared with any other soil properties, imply the predominant role of hydrophobic interactions with soil organic matter in the sorption of the SAs. Both *K*_f_ and *K*_d_ of SMX were negatively correlated with soil pH (*p* < 0.01), indicating hydrogen bonding might play a more important role in its sorption to the soils (particularly in the acidic soils) compared with the other three SAs. A previous study with 13 soils with pH ranging from 5.3 to 8.7 also reported a negative correlation of *K*_f_ with soil pH for SMX [10]. Antibiotic sorption may also be affected by soil texture [45]. The most significant negative correlation of sand content with *K*_f_ and *K*_d_ was observed for SDZ. Positive correlations of clay content with *K*_f_ and *K*_d_ were found for both the most strongly sorbing SCP and the most weakly sorbing SDZ (*p* < 0.01). Similarly, a number of previous studies in acidic soils have reported positive correlations of *K*_d_ and/or *K*_f_ for SAs with OC, CEC, and clay content but negative correlations with sand content [14,31,32,46].

*K*_f_ and/or *K*_d_ of the TCs showed a positive correlation with OC (*p* < 0.01), indicating that their interactions with SOM through hydrophobic interactions (e.g., π-π electron donor–acceptor interaction and van der Waals attractions) were important sorption mechanisms, as also reported previously [47,48,49]. Notably, given the similar correlation coefficients of *K*_f_ or *K*_d_ with OC for the three TCs, CTC’s much higher *K*_f_ and *K*_d_ than those of OTC and TC (Table 2) can be attributed to the highest log*K*_ow_ (a key parameter of a hydrophobic antibiotic) of CTC (Table 1). On the other hand, *K*_f_ of all the three TCs were negatively correlated with soil pH, implying that cation exchange also played a key role in the sorption of the TCs (*p* < 0.01). Despite the inconsistent relationships between *K*_f_/*K*_d_ and CEC observed among different TCs (Table 3), it can be inferred that cation exchange between soil surfaces and the protonated amine groups of TCs was the main sorption mechanism at pH lower than their p*K*_a1_ [50,51]. Moreover, *K*_d_ of the least strongly sorbing TC showed a strong negative correlation with sand content, while showing positive correlations with clay and silt content as well as CEC (*p* < 0.01). The observed texture effect agrees with a previous finding that both *K*_f_ and *K*_d_ of OTC in a clay loam soil were higher than in a loamy sand soil [24].

Overall, the main soil properties influencing the sorption of the SAs were OC and CEC, while key influential soil properties for TC sorption were OC and pH. Apparently, the effect of soil texture on antibiotic sorption was inconsistent and antibiotic specific.

### 3.4. Model Development and Validation

**Figure 1:**
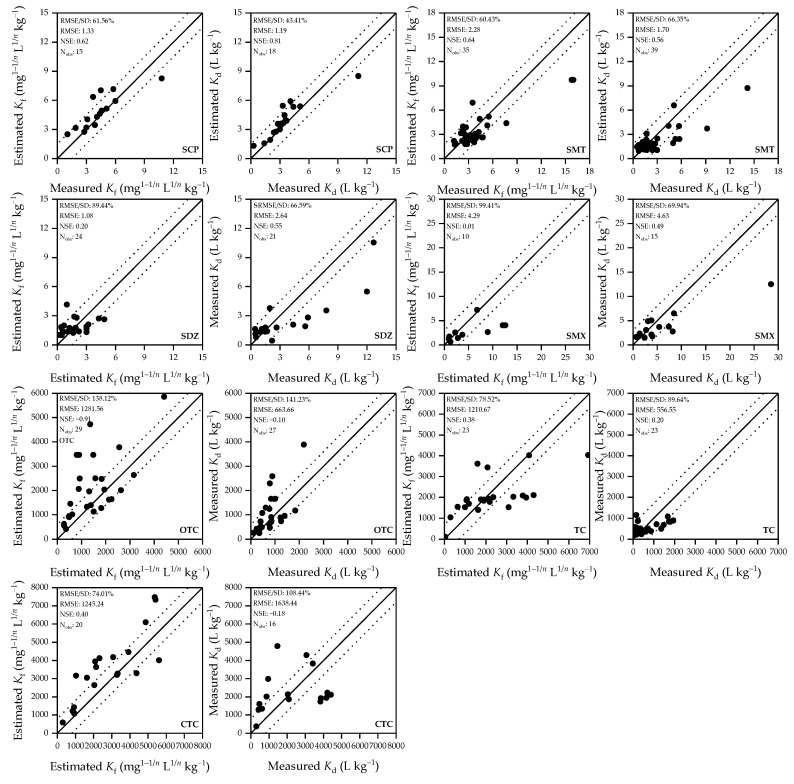
Measured *K*_f_ and *K*_d_ in sub-datasets “a” and “b” against estimated *K*_f_ and *K*_d_ from the pedotransfer functions. The solid line represents a perfect model fit (1:1 line), and the dashed lines represent a difference of one order of magnitude, which indicate satisfactorily estimated values.

Pedotransfer functions developed from sub-datasets “A” and “B” are presented in Table 4, and the results of model validation with sub-datasets “a” and “b” are shown in Figure 1. The models for all target antibiotics yielded good estimations of both *K*_f_ and *K*_d_. For sub-datasets “A” and “B”, RMSE of the pedotransfer functions for *K*_f_ ranged from 0.39 to 2.01 and from 612.94 to 1340.46 for the SAs and the TCs, respectively; whereas, their RMSE/SD ratios for all target antibiotics fell within a narrow range (56.2–77.2%). Irrespective of antibiotic type, NSE of the pedotransfer functions for *K*_f_ ranged from 0.40 to 0.69 for sub-dataset “A”, reflecting good model performances. The values of soil properties in sub-datasets “a” and “b” were mostly within the ranges of sub-datasets “A” and “B”. For sub-dataset “a”, RMSE of the pedotransfer functions for *K*_f_ ranged from 1.08 to 4.29 and from 1210.67 to 1281.56 for the SAs and the TCs, respectively; their RMSE/SD ratios ranged from 60.4% to 99.4% and from 74.0% to 138.1% for the SAs and the TCs, respectively. Regardless of antibiotic type, NSE of the pedotransfer functions for *K*_f_ ranged from –0.91 to 0.64 for sub-dataset “a”, which indicated good estimations and were only slightly lower than those for sub-dataset “A”. The pedotransfer functions for *K*_f_ were thus validated by their satisfactory performances observed for sub-dataset “a”. Similarly, the pedotransfer functions developed for *K*_d_ with sub-dataset “B” were also satisfactorily validated with sub-dataset “b”.

In addition to basic soil properties, differences in experimental methods for soil characterization (especially for soil texture and CEC), maximal initial concentration (*C*_imax_), and solid/liquid ratio (*SLR*) for batch sorption test, may be partly responsible for the variations in measured affinity coefficients (*K*_f_, *K*_d_), but these experimental parameters have not been used as input variable(s) for multiple linear regression analysis in previous studies [52]. Moreover, the inclusion of parameters of antibiotic species in multiple linear regression analysis may also help develop better models for estimating affinity coefficients [31]. In this study, some of these parameters, including *C*_imax_, *SLR*, and percentage of antibiotic form(s) at a given pH (*α*^+^, *α*^0^, or *α*^–^, representing cationic, neutral/zwitterion, or anionic species, respectively), were considered as additional independent variables, and eight models with better performances in estimating *K*_f_ and/or *K*_d_ were thus developed for all the target antibiotics except SMT (Table 5). Performance of the improved models for SCP was slightly better (as indicated by a 0.3% and 0.6% increase in *r*^2^ for *K*_f_ and *K*_d_, respectively), and moderately better model performances (as indicated by 2.5–7.2% increase in *r*^2^) were achieved for SDZ, SMX, and the TCs. This improvement in model performance for the three SAs can be explained by the dependence of relative importance of various sorption mechanisms on antibiotic species distribution at a given pH. As for the TCs, these improvements can be attributed to the inclusion of not only species distribution parameters, but also *SLR* and/or *C*_imax_, which can reflect the non-linear sorption behavior of TCs. This is in line with the finding that the smaller the SLR, the fewer the sorption sites, as a result of more rapid saturation of the sorption sites with increasing pesticide concentration [52]. Similarly, the lowering of *SLR* from 1:10 to 1:50 was found to cause decreases in sorption of TCs by 75% and 43% for alfisol and ultisol, respectively [40]. It should be noted that this study was not able to obtain improved models for SMT, and improved models were successfully built for estimating either *K*_f_ or *K*_d_ (not both) for four antibiotics (SDZ, SMX, OTC, and CTC) and both *K*_f_ and *K*_d_ for the other two antibiotics (SCP and TC). Apparently, the improvements in model performance achieved by incorporating additional non-edaphic variable(s) were limited, and therefore the validation of these models was not conducted further.

**Figure 2:**
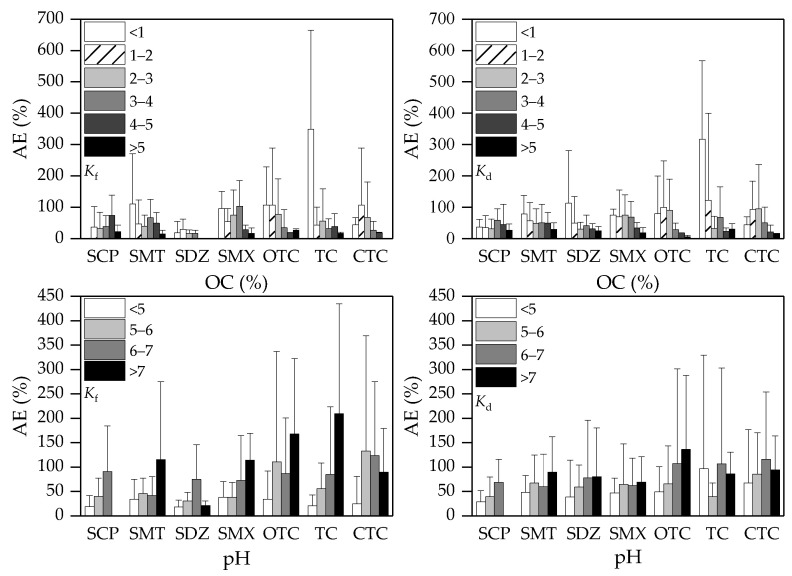
Absolute error (AE) distributions of *K*_f_ and *K*_d_ estimation across different OC and pH ranges in sub-datasets “A” and “B” using the pedotransfer equations given in Table 4.

Soil OC and pH are the two most useful edaphic variables that can be used to estimate *K*_f_ and *K*_d_ of a specific SA or TC antibiotic in dissimilar soils. The effects of OC and pH variation on the performance of the pedotransfer functions (Table 4) were evaluated using sub-datasets “A” and “B” in terms of AE, and the AE values obtained for different OC or pH ranges are shown in Figure 2. It was found that the soils with higher OC content showed lower AE in estimating *K*_f_ and *K*_d_, with the lowest AE (19.8% and 22.0% for *K*_f_ and *K*_d_, respectively) being observed in the soils with OC content greater than 5%. Since SOM was the most influential soil property for the sorption of SAs and TCs (Table 3), a higher OC content can lead to a greater ability of SOM to estimate affinity coefficients. Contrastingly, a higher soil pH was associated with a poorer model performance (i.e., a higher AE), which can be attributed to a decreased importance of strong sorption mechanisms (e.g., cation exchange and electrostatic attraction) but an increased importance of weak sorption mechanisms (e.g., π-π interaction, and van der Waals forces) at an increased pH. For sub-datasets “a” and “b”, similar AE distributions across different OC or pH ranges were found for SAs, TC, and CTC (Figure S1). Nevertheless, AE of OTC estimation using sub-datasets “a” and “b” showed opposite trends with increasing OC and pH, which might be caused by the limitation of the small observation number in sub-datasets “a” and “b” and the generally lower NSE for OTC than the other antibiotics. Apparently, the pedotransfer functions of OTC did not perform as well as those for the other antibiotics (Figure 1). It is expected that the pedotransfer functions developed in this study can give better estimation of *K*_f_ and *K*_d_ for soils with higher OC and lower pH.

## 4. Discussion

### 4.1. Governing Factors and Mechanisms of Antibiotic Sorption

Soil pH, OC, CEC, and texture are readily available parameters that may be correlated with sorption capacity (i.e., *K*_f_ and/or *K*_d_) of antibiotics [10,11,12,31,32,41,53,54,55]. Among these edaphic parameters, OC showed the highest positive correlation with either *K*_f_ or *K*_d_ for most antibiotics investigated in this study, implying the dominant role of hydrophobic interactions (e.g., π-π electron donor–acceptor interaction, and van der Waals interactions) in sorption to soil organic matter [42]. In addition, other sorption mechanisms include hydrogen bonding of antibiotics with hydroxyl groups on soil organic matter, particularly in acidic soils [17,22], and electrostatic interactions (e.g., cation exchange, surface complexation with potential contribution of cation bridging, charge transfer, and ligand exchange) of antibiotics with negatively charged surfaces [10,23]. It should be noted that electrostatic forces are stronger than hydrophobic interactions [37,56].

The SAs are acidic and largely uncharged, or negatively charged at natural soil pH (e.g., ≥3.7 in this study), as indicated by p*K*_a1_ ≤ 2.1 (Table 1). It has been well recognized that hydrophobic partitioning of soil organic matter is a main mechanism for the sorption of SAs [32,57,58,59]. Hydrophobic interactions were more important sorption mechanisms for the SAs than for the TCs, as reflected by the observed higher *r* values between *K*_f_ or *K*_d_ and OC for the SAs (Table 3). In addition to OC, CEC was another predominant edaphic parameter affecting the sorption of the SAs, probably as a result of the exchange of cationic SA species on negatively charged sites of clay mineral surfaces or organic matter, and surface complexation of anionic SA species on the edges of layered clay minerals and radical fragments of humus via cation bridges [16]. The important role of hydrogen bonding in sorption was demonstrated by FTIR spectrum analysis for SDZ and SMX [60].

The TCs, which are basic (represented by high p*K*_a3_) and thus have higher contents of cation form than the SAs at natural soil pH, could show more significant non-hydrophobic interactions (e.g., cation exchange, ligand exchange, surface complexation, and H bonding) in soils [61]. The negative correlations of soil pH with *K*_f_ for the TCs observed in this study agree with the results of many previous studies [13,17]. For instance, in a study with 63 soils, a negative correlation of *K*_f_ with soil pH was observed for OTC (*p* < 0.01) but not for CTC [13]. Similarly, negative correlations with *K*_Te_ of the Temkin model with soil pH were reported for TC [17]. It should be noted that the predominant sorption mechanism of TCs may vary with soil pH. For instance, cation exchange might be the predominant sorption mechanism of TC in alkaline soils while hydrophobic interactions might be its primary sorption mechanism in acidic soils [11,17].

Overall, SOM dominates the sorption of SAs and TCs through hydrophobic interactions with neutral/zwitterion species of the antibiotics [17,31,32,58,62], hydrogen bonding of its protonated sites with polar groups of the antibiotics [19], and forming complexes with the antibiotics [7]. Moreover, both SA and TC antibiotics can covalently attach SOM [15,17,63]. Clay minerals may have a positive effect on antibiotic sorption due to their greater amounts of negative charge, larger surface area and higher CEC, as well as more preferential association with organic matter, while sand often shows a negative effect [13,14,17]. In the low hydrophobicity range, clay minerals in soils may play a significant role in sorption through cation exchange and cation bridging [64,65,66]. Notably, cation bridges, which can form on the surfaces of both soil minerals (aluminosilicates, metal oxides) and organic matter [67,68], may play a more important role in complexation with anionic species of SAs, compared with TCs which are less negatively charged, particularly in acidic soils. The relative importance of different mechanisms is dependent on the physicochemical properties of soils and antibiotics, and environmental factors (e.g., pH, ionic strength, organic matter, and temperature) [69]. A decrease in soil solution pH can lead to an increased proportion of the cationic species of SAs and TCs and thus an enhanced sorption of antibiotics via cation exchange [66,70,71,72].

### 4.2. Model Performance

The pedotransfer functions established in this study are very useful, as most of them (11 of 14) could explain more than 50% of the variance of *K*_f_ or *K*_d_ (Table 4). The multiple linear regression analysis indicated that OC was the only edaphic variable commonly included in all the established functions except that for *K*_d_ of TC, with explanation of the variance being 58.9% (*K*_f_) and 67.3% (*K*_d_) for SCP, 56.5% (*K*_f_) and 51.0% (*K*_d_) for SMT, 20.4% (*K*_f_) and 46.4% (*K*_d_) for SDZ, 53.5% (*K*_f_) and 38.1% (*K*_d_) for SMX, 4.9% (*K*_f_) and 44.4% (*K*_d_) for OTC, 33.5% (*K*_f_) for TC, and 49.8% (*K*_f_) and 32.9% (*K*_d_) for CTC. pH was the secondary edaphic variable, which was included in 8 of the 14 models and could achieve a maximum explanation (55.7%) of the variance of *K*_f_ for OTC. CEC could explain only the variance of *K*_f_ for SMX and the variance of *K*_f_ and *K*_d_ for TC by smaller percentages, reflecting the lesser ability of CEC to estimate antibiotic sorption than OC and pH. The variance of *K*_f_ for SDZ and SMX and the variance of *K*_d_ for TC and CTC could be partly explained by selected soil textural parameters (e.g., sand, silt, and clay content).

In most previous studies, linear models were established based on batch sorption experiments in a suit of different soils for a single or a few antibiotics, such as sulfachloropyridazine [24,30,32], sulfadiazine [31], sulfamethazine [30,32], oxytetracycline [2,13,24], chlortetracycline [13], and tylosin [24]. The performance of selected published models was evaluated using sub-datasets “a” and “b” and the results are shown in Table 6. For SCP and SMT, RMSE, RMES/SD and NSE of published models were very close to those of our models (Figure 1); however, for SDZ and OTC, the performances of published models were poorer than those of our models (Figure 1). This result could be explained by the following: on the one hand, SOM played a more dominant role in the sorption of SCP and SMT compared with SDZ and OTC; on the other hand, in addition to SOM, the sorption of SDZ and OTC was also affected by soil pH and texture, which were more effectively represented in our models. It should be noted that the data used for the development of these published models were limited and regionally constrained. For instance, the models for SCP and SDZ were established with soils showing OC and pH in the range of 1.1–10.9 and 3.7–6.2, respectively [31,32]; the model for SMT was established with soils showing OC in the range of 0.1–3.8 [56]; and the model for OTC was established with soils showing OC and CEC in the range of 1.1–10.9 and 3.8–30.31, respectively [13]. Apparently, these published models were applicable only to a narrower range of soils compared with our models (Table S1).

The pedotransfer functions developed for two major groups of antibiotics in this study are simple and can be applied in environmental risk assessment of antibiotics in soils. The different effects of inclusion of non-edaphic variable(s) on model performance among antibiotics and affinity coefficients indicated that more than one sorption mechanism might dominate and the relative importance of one mechanism over another depended on, in addition to soil properties, antibiotic species and environmental conditions (e.g., pollution level and soil to water ratio). Given the complex relationships of affinity coefficients with varying properties/parameters, some previous studies employed machine learning approaches (artificial neural network, random forest, and support vector machine) to develop nonlinear models for antibiotics (together with non-antibiotic pharmaceuticals), and the best performance was achieved by a random forest-based model using antibiotic and soil properties as the independent variable(s) [25,73,74]. Notably, the random forest model can be utilized to reveal the relative importance order of variables and thus may help select the top contributing variables for the development of new models [74]. Compared with traditional regression models, machine learning models are more complex in nature and less transparent for users. From a regulatory perspective, simple and transparent methods would be preferred.

In addition, the applicability of our model for SMT was tested for sulfadimethoxine (SDM), which is an SA antibiotic that has similar physico-chemical properties to SMT [75]. Results showed that it performed well in predicting *K*_f_ of SDM, with RMSE, RMSE/SD, and NSE being 2.23, 34.1%, and 0.88, respectively (Table S10). Nevertheless, this potential for a wider application needs to be verified with more data in future studies.

### 4.3. Future Perspectives

It would be costly and time consuming to experimentally measure the sorption parameters of all antibiotics in all soils in an area of interest or globally. Given the complexities of antibiotic–soil interactions, future efforts to improve the predictive performance of new models should be directed to the following: generation of a bigger high-quality dataset of antibiotic sorption and associated soil properties with standard experimental protocols and development of conversion methods for results obtained under varying non-standard experimental conditions (e.g., initial aqueous antibiotic concentrations, soil to solution ratio, and solution electrolyte composition, temperature, and pH) and analytical methods; comparison of traditional regression models and machine learning models with independent datasets to identify their suitability for different antibiotics and soils; and the building of different model options that can meet varying requirements of accuracy.

## 5. Conclusions

A dataset of sorption parameters for 4 SAs and 3 TCs in soils collected from the literature was built, and key soil factors (OC, pH and CEC) affecting antibiotic sorption were identified using correlation analysis. Linear pedotransfer functions for estimating *K*_f_ and *K*_d_ were successfully established by multiple linear regression analysis and were satisfactorily validated. The new pedotransfer functions developed in this study can be used as an easy tool for environmental risk assessment, prioritization of antibiotics and identification of vulnerable soils in an area of interest, which could help develop mitigation measures to minimize the adverse impacts of antibiotic pollutants on human and environmental health.

## Figures and Tables

**Table 1:** Physicochemical properties of the target antibiotics.

Antibiotic	Physical-Chemical Properties		Chemical Structure	Species Distribution ^f^
SCP	Molecular formula	C_10_H_9_ClN_4_O_2_S	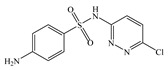	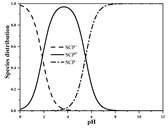
	Solubility (mg L^−1^)	7000.00 ^c^		
	M_w_ (g mol^−1^)	284.72		
	log*K*_ow_	–0.80 ^a^		
	p*K*_a1_	1.87 ^a^		
	p*K*_a2_	5.45 ^a^		
SMT	Molecular formula	C_12_H_14_N_4_O_2_S	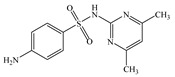	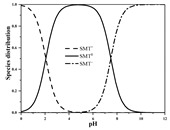
	Solubility (mg L^−1^)	1500.00 ^a^		
	M_w_ (g mol^−1^)	278.34		
	log*K*_ow_	0.14 ^a^		
	p*K*_a1_	2.07 ^a^		
	p*K*_a2_	7.49 ^a^		
SDZ	Molecular formula	C_10_H_10_N_4_O_2_S	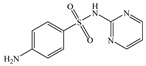	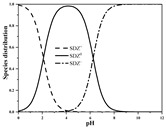
	Solubility (mg L^−1^)	77.00 ^a^		
	M_w_ (g mol^−1^)	250.30		
	log*K*_ow_	−1.05 ^a^		
	p*K*_a1_	2.10 ^a^		
	p*K*_a2_	6.28 ^a^		
SMX	Molecular formula	C_10_H_11_N_3_O_3_S	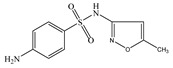	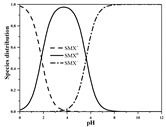
	Solubility (mg L^−1^)	370.00 ^b^		
	M_w_ (g mol^−1^)	253.28		
	log*K*_ow_	0.89 ^b^		
	p*K*_a1_	1.83 ^b^		
	p*K*_a2_	5.62 ^b^		
OTC	Molecular formula	C_22_H_24_N_2_O_9_	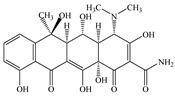	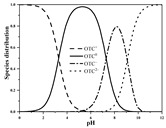
	Solubility (mg L^−1^)	1000.00 ^c^		
	M_w_ (g mol^−1^)	460.40		
	log*K*_ow_	–0.12 ^c^		
	p*K*_a1_	3.30 ^c^		
	p*K*_a2_	7.30 ^c^		
	p*K*_a3_	9.10 ^c^		
TC	Molecular formula	C_22_H_24_N_2_O_8_	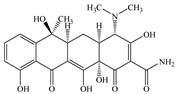	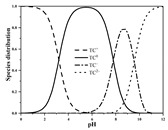
	Solubility (mg L^−1^)	231.00 ^d^		
	M_w_ (g mol^−1^)	444.43		
	log*K*_ow_	−1.37 ^d^		
	p*K*_a1_	3.20 ^d^		
	p*K*_a2_	7.80 ^d^		
	p*K*_a3_	9.60 ^d^		
CTC	Molecular formula	C_22_H_23_ClN_2_O_8_	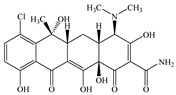	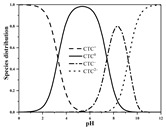
	Solubility (mg L^−1^)	4120.00 ^e^		
	M_w_ (g mol^−1^)	479.00		
	log*K*_ow_	2.07 ^e^		
	p*K*_a1_	3.30 ^e^		
	p*K*_a2_	7.44 ^e^		
	p*K*_a3_	9.27 ^e^		

Note: ^a^ [5]; ^b^ [35]; ^c^ [36]; ^d^ [30]; ^e^ [37]; ^f^ [38].

**Table 2:** Statistical characteristics of sorption parameters of individual antibiotics in the soils.

Antibiotic	Parameter	Statistics				
		Max	Min	Mean	Median	N_obs_ *
SCP	*K*_f_ (mg^1−1/*n*^ L^1/*n*^ kg^−1^)	15.33	0.60	5.25	4.60	83
	*n*	2.98	0.90	1.18	1.20	83
	*K*_d_ (L kg^−1^)	23.10	0.30	4.42	3.25	98
SMT	*K*_f_ (mg^1−1/*n*^ L^1/*n*^ kg^−1^)	16.00	0.13	3.35	2.67	142
	*n*	4.00	0.41	1.20	1.20	142
	*K*_d_ (L kg^−1^)	14.20	0.11	2.45	1.62	153
SDZ	*K*_f_ (mg^1−1/*n*^ L^1/*n*^ kg^−1^)	4.86	0.45	1.65	1.45	77
	*n*	2.17	0.28	1.00	1.00	77
	*K*_d_ (L kg^−1^)	12.70	0.09	2.05	1.40	104
SMX	*K*_f_ (mg^1−1/*n*^ L^1/*n*^ kg^−1^)	12.60	0.133	2.76	2.00	59
	*n*	2.38	0.48	1.28	1.20	59
	*K*_d_ (L kg^−1^)	28.50	0.02	2.49	1.40	72
OTC	*K*_f_ (mg^1−1/*n*^ L^1/*n*^ kg^−1^)	5110.00	74.00	1901.52	1814.00	133
	*n*	6.25	0.30	2.09	1.85	126
	*K*_d_ (L kg^−1^)	2191.00	16.76	692.59	601.17	121
TC	*K*_f_ (mg^1−1/*n*^ L^1/*n*^ kg^−1^)	6928.09	0.28	1707.47	1640.61	107
	*n*	6.85	0.39	2.10	2.13	107
	*K*_d_ (L kg^−1^)	1940.89	10.06	481.53	362.30	90
CTC	*K*_f_ (mg^1−1/*n*^ L^1/*n*^ kg^−1^)	8176.99	302.00	3220.16	3131.56	93
	*n*	4.00	0.43	2.20	2.33	93
	*K*_d_ (L kg^−1^)	4473.20	147.08	1596.77	1219.21	88

* Number of reported observations.

**Table 3:** Pearson correlation coefficients (*r*) of sorption parameters with soil properties.

Antibiotic	Soil Property	*K*_f_	*n*	*K*_d_
SCP	pH	0.071	0.248 *	0.080
	OC	0.772 **	–0.120	0.631 **
	CEC	0.338 **	0.215 *	0.200 *
	Sand	–0.207 *	–0.122	–0.170
	Silt	0.075	0.081	–0.080
	Clay	0.310 **	0.107	0.372 **
SMT	pH	–0.009	0.056	0.011
	OC	0.754 **	–0.172 *	0.717 **
	CEC	0.409 **	0.035	0.269 **
	Sand	–0.137	–0.211 *	0.040
	Silt	0.124	0.179 *	–0.062
	Clay	0.101	0.163 *	0.014
SDZ	pH	0.068	0.160	0.267 **
	OC	0.364 **	–0.151	0.686 **
	CEC	–0.119	0.088	0.353 **
	Sand	–0.439 **	–0.263 *	–0.415 **
	Silt	0.287 *	0.195	0.203 *
	Clay	0.380 **	0.195	0.442 **
SMX	pH	–0.381 **	0.082	–0.389 **
	OC	0.738 **	–0.180	0.626 **
	CEC	0.435 **	0.026	0.270 *
	Sand	0.052	0.109	–0.008
	Silt	–0.214	–0.028	–0.063
	Clay	0.235	–0.150	0.119
OTC	pH	–0.749 **	–0.612 **	–0.268 **
	OC	0.587 **	0.216 *	0.670 **
	CEC	–0.488 **	–0.490 **	0.146
	Sand	0.350 **	0.374 **	–0.030
	Silt	–0.313 **	–0.293 **	0.011
	Clay	–0.174 *	–0.252 **	0.048
TC	pH	–0.570 **	–0.727 **	0.283 *
	OC	0.585 **	0.135	0.151
	CEC	–0.093	–0.603 **	0.480 **
	Sand	0.186 *	0.474 **	–0.704 **
	Silt	–0.175	–0.463 **	0.457 **
	Clay	–0.104	–0.265 **	0.669 **
CTC	pH	–0.457 **	–0.737 **	–0.149
	OC	0.684 **	0.302 **	0.419 **
	CEC	–0.240 *	–0.672 **	0.073
	Sand	0.127	0.576 **	–0.061
	Silt	–0.081	–0.417 **	–0.049
	Clay	–0.104	–0.426 **	0.226 *

Note: * and ** represent significance at the 0.05 and 0.01 probability level, respectively.

**Table 4:** Statistical characteristics of sorption parameters of individual antibiotics in the soils.

Antibiotic	Pedotransfer Function	*r* ^2^	RMSE (RMSE/SD)	NSE	N_obs_ ^1^
SCP	$K_{f}=4.198+1.666\mathrm{OC}-0.735\mathrm{pH}$	0.616 ** ^2^	2.01(61.1%)	0.63	68
	$K_{d}=2.769+1.668\mathrm{OC}-0.689\mathrm{pH}$	0.689 **	2.34 (55.1%)	0.70	80
SMT	$K_{f}=0.820+0.818\mathrm{OC}$	0.565 **	1.24 (65.6%)	0.57	107
	$K_{d}=0.099+0.789\mathrm{OC}$	0.510 **	1.58 (69.6%)	0.52	114
SDZ	$K_{f}=1.951+0.239\mathrm{OC}-0.018\mathrm{Sand}$	0.380 **	0.39 (77.2%)	0.40	53
	$K_{d}=-0.480+0.484\mathrm{OC}+0.201\mathrm{pH}$	0.507 **	0.73 (69.4%)	0.52	83
SMX	$K_{f}=4.717+0.464\mathrm{OC}-0.565\mathrm{pH}+0.006\mathrm{CEC}-0.027\mathrm{Silt}$	0.666 **	1.07 (56.2%)	0.69	49
	$K_{d}=3.208+0.519\mathrm{OC}-0.457\mathrm{pH}$	0.525 **	0.88 (67.2%)	0.54	57
OTC	$K_{f}=4428.177+330.323\mathrm{OC}-543.318\mathrm{pH}$	0.606 **	851.94 (62.2%)	0.61	104
	$K_{d}=182.875+239.030\mathrm{OC}$	0.444 **	328.77 (74.2%)	0.45	94
TC	$K_{f}=2740.451+215.512\mathrm{OC}-363.881\mathrm{pH}+3.331\mathrm{CEC}$	0.509 **	612.94 (68.8%)	0.53	84
	$K_{d}=274.636+1.151\mathrm{CEC}-5.607\mathrm{Sand}+14.741\mathrm{Clay}$	0.607 **	179.22 (61.3%)	0.62	67
CTC	$K_{f}=2345.591+1205.573\mathrm{OC}-281.455\mathrm{pH}$	0.524 **	1340.46 (68.0%)	0.54	73
	$K_{d}=-588.94+561.887\mathrm{OC}+38.582\mathrm{Clay}$	0.371 **	811.11 (78.2%)	0.39	72

^1^ Number of reported observations. ^2^ ** represents significance at the 0.01 probability level.

**Table 5:** Improved models by inclusion of additional independent variable(s) for estimating *K*_f_ and *K*_d_ of antibiotics in soils (based on sub-datasets “A” and “B”).

Antibiotic	Pedotransfer Function	R^2^
SCP	$K_{f}=-0.807+1.657\mathrm{OC}+2.023\alpha^{0}$	0.619 **
	$K_{d}=-1.149+1.657\mathrm{OC}+183.089\alpha^{+}$	0.695 **
SDZ	$K_{d}=0.980+0.398\mathrm{OC}+0.200\mathrm{Clay}-0.949\alpha^{0}$	0.579 **
SMX	$K_{d}=1.652+0.485\mathrm{OC}-1.91\alpha^{–}$	0.552 **
OTC	$K_{f}=4595.375-701.271\mathrm{pH}+397.550\mathrm{OC}+16589.89\mathrm{SLR}+5204.082\alpha^{–}$	0.647 **
TC	$K_{f}=2863.937+288.648\mathrm{OC}-265.391\mathrm{pH}-20202.101\mathrm{SLR}$	0.545 **
	$K_{d}=325.965+51.147\mathrm{OC}-6.099\mathrm{Sand}+15.714\mathrm{Clay}-1058.792\alpha^{+}$	0.633 **
CTC	$K_{f}=-256.377+1219.651\mathrm{OC}+6.639C_{\mathrm{imax}}$	0.549 **

Note: ** represents significance at the 0.01 probability level.

**Table 6:** Performance of previously published models for sub-datasets “a” and “b”.

Antibiotic	Pedotransfer Function	*r* ^2^	Origin	N_obs_ ^1^	RMSE (RMSE/SD)	NSE	Reference
SCP	$K_{f}=8.810+1.967\mathrm{OC}-2.028\mathrm{pH}$	0.829 ** ^2^	Galicia (Spain)	50	1.35 (62.4%)	0.61	[32]
SMT	$K_{d}=0.38+0.81\mathrm{OC}$	0.92 **	Iowa (USA)	5	1.62 (63.0%)	0.60	[56]
SDZ	$K_{f}=3.493+0.780\mathrm{OC}-0.819\mathrm{pH}$	0.675 **	Galicia (Spain)	50	1.81 (149.6%)	−1.20	[31]
OTC	$K_{f}=96.924+701.607\mathrm{OC}+8118.902{\mathrm{CEC}}^{-1}$	0.349 **	Galicia (Spain)	63	2052.63 (221.87%)	−3.90	[13]

^1^ Number of reported observations used to evaluate the performance of previously published models. ^2^ ** represents significance at the 0.01 probability level.

## Data Availability

Not applicable.

## Supplementary Materials

The following supporting information can be downloaded at: https://www.mdpi.com/article/10.3390/ijerph192416771/s1, Figure S1: Absolute errors (AE) distributions of *K*_f_ and *K*_d_ estimation across different OC and pH ranges in sub-datasets “a” and “b” using the pedotransfer equations given in Table 4; Table S1: Statistical characteristics of basic properties of the soils used for individual target antibiotics; Table S2: SCP sorption parameters and associated soil properties; Table S3: SMT sorption parameters and associated soil properties; Table S4: SDZ sorption parameters and associated soil properties; Table S5: SMX sorption parameters and associated soil properties; Table S6: OTC sorption parameters and associated soil properties; Table S7: TC sorption parameters and associated soil properties; Table S8: CTC sorption parameters and associated soil properties; Table S9: pH distribution of the soils in sub-datasets “A” (for *K*_f_) and “B” (for *K*_d_) and main antibiotic species in soil water; Table S10: SDM sorption parameters, associated soil properties, and the performance of the model established for SMT in this study. References [76,77,78,79,80,81,82,83,84,85,86,87,88,89,90,91,92,93,94,95,96,97,98,99,100,101,102,103,104,105,106,107,108,109,110,111,112,113,114,115,116,117,118,119,120,121,122,123,124,125,126,127,128,129,130,131,132,133,134,135,136,137,138,139,140,141,142,143,144,145,146] are cited in the supplementary materials.
