# Supplementary material for: New Models for Estimating the Sorption of Sulfonamide and Tetracycline Antibiotics in Soils
Source: Int J Environ Res Public Health. 2022 Dec 14;19(24):16771. doi: 10.3390/ijerph192416771 (PMC9778684; doi:10.3390/ijerph192416771)
Supplement: Supplementary file 1 [file ijerph-19-16771-s001.zip › ijerph-2060552-supplementary.pdf]

Supplementary Material

New Models for Estimating the Sorption of Sulfonamide and Tetracycline Antibiotics in Soils

Jinsheng Hu [1,2,3], Xiangyu Tang [1,3,*], Minghui Qi [3] and Jianhua Cheng [3]

[1] Institute of Mountain Hazards and Environment, Chinese Academy of Sciences, Chengdu 610041, China
[2] University of Chinese Academy of Sciences, Beijing 100049, China
[3] State Key Laboratory of Subtropical Silviculture, Zhejiang A&F University, Hangzhou 311300, China
* Correspondence: xytang@imde.ac.cn

Number of figures: 1

Number of tables: 10

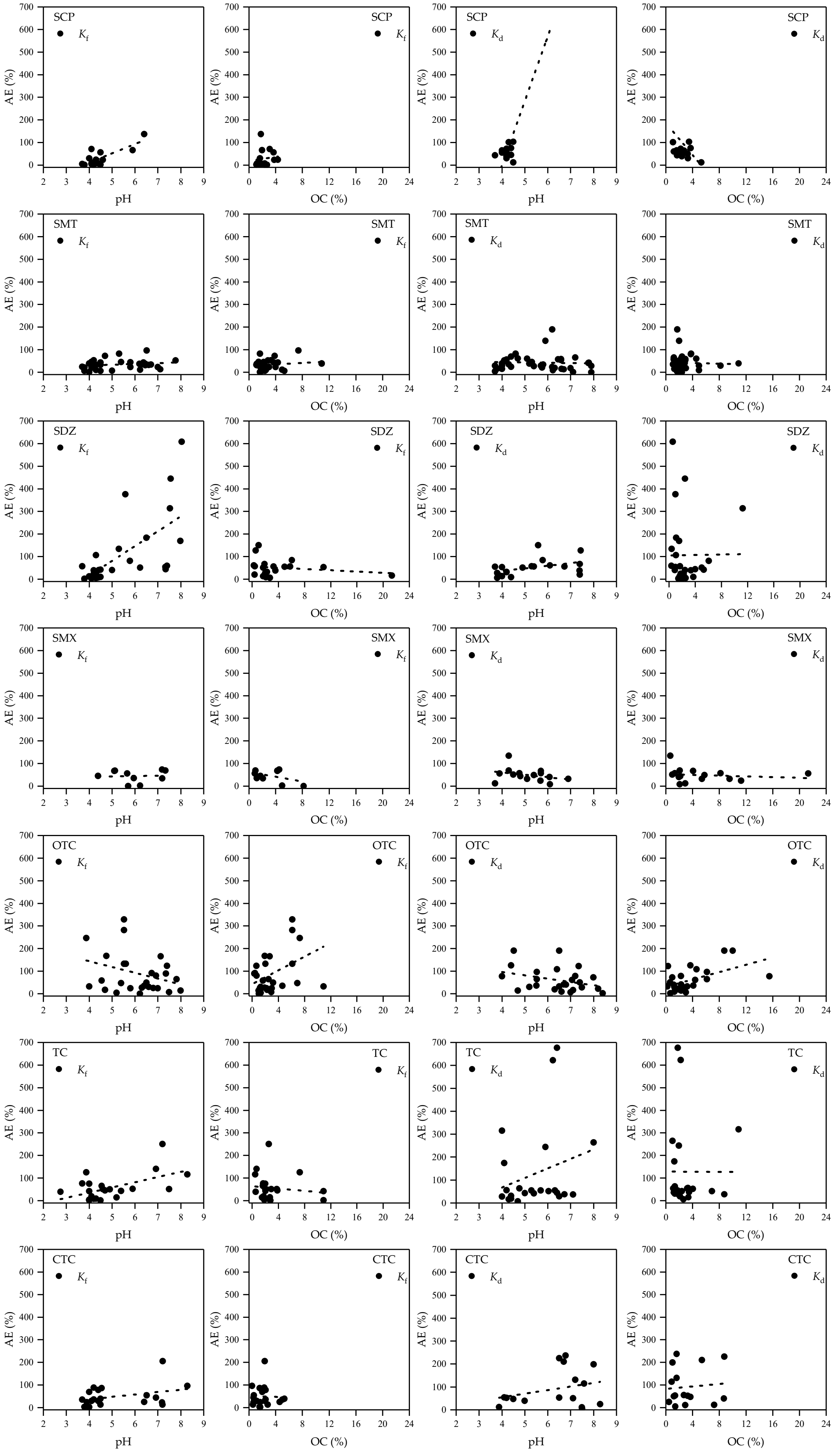

Figure S1. Absolute errors (AE) distributions of K_f and K_d estimation across different OC and pH ranges in sub-datasets "a" and "b" using the pedotransfer equations given in Table 4.

Table S1. Statistical characteristics of basic properties of the soils used for individual target antibiotics.

Antibiotic	Parameter	Statistics				
		**Max**	**Min**	**Mean**	**Median**	**$N_{obs.}$ ***
SCP	pH	6.90	3.70	4.85	4.50	100
	OC (%)	10.90	0.67	3.08	2.46	100
	CEC (mmol kg^{-1})	356.00	27.60	98.62	65.52	100
	Sand (%)	88.00	9.00	49.88	53.50	100
	Silt (%)	62.00	2.00	27.21	22.00	100
	Clay (%)	68.40	4.00	22.91	20.00	100
SMT	pH	8.60	3.70	5.47	5.40	155
	OC (%)	10.90	0.10	2.78	2.20	155
	CEC (mmol kg^{-1})	356.00	15.78	101.85	69.10	150
	Sand (%)	100.00	2.40	56.11	56.00	146
	Silt (%)	82.90	0.00	25.12	21.00	146
	Clay (%)	62.00	0.00	18.96	19.00	155
SDZ	pH	8.60	3.70	5.10	4.50	108
	OC (%)	21.34	0.16	2.67	2.00	108
	CEC (mmol kg^{-1})	740.00	7.40	97.71	61.90	105
	Sand (%)	91.10	1.40	49.34	55.00	108
	Silt (%)	89.70	1.80	27.72	21.90	108
	Clay (%)	68.40	1.20	22.58	20.00	108
SMX	pH	8.60	3.70	5.89	5.70	80
	OC (%)	21.34	0.08	2.85	2.02	80
	CEC (mmol kg^{-1})	538.10	3.40	157.22	155.00	79
	Sand (%)	100.00	2.40	38.74	35.50	80
	Silt (%)	82.90	0.00	39.78	40.00	80
	Clay (%)	68.40	0.00	21.43	18.30	80
OTC	pH	8.98	3.50	5.66	5.52	149
	OC (%)	15.49	0.18	2.35	1.88	158
	CEC (mmol kg^{-1})	600.00	8.20	123.79	105.10	154
	Sand (%)	94.90	1.18	47.35	51.50	133
	Silt (%)	79.19	3.80	31.49	27.00	133
	Clay (%)	77.16	1.20	21.83	19.95	146
TC	pH	8.98	2.75	5.49	5.10	119
	OC (%)	10.92	0.12	2.51	2.06	119
	CEC (mmol kg^{-1})	600.00	19.90	116.98	86.20	119
	Sand (%)	82.26	7.70	47.47	53.00	115
	Silt (%)	65.80	10.30	31.01	26.00	115
	Clay (%)	48.90	1.20	21.53	20.00	117
CTC	pH	9.40	3.70	5.51	5.00	104
	OC (%)	8.78	0.30	2.30	2.00	104
	CEC (mmol kg^{-1})	600.00	38.80	113.08	80.30	99
	Sand (%)	81.00	6.10	46.98	52.00	96
	Silt (%)	78.20	10.30	31.02	24.20	96
	Clay (%)	49.60	0.07	21.93	20.00	98

* Number of reported observations.

Table S2. SCP sorption parameters and associated soil properties.

No.	Origin	Soil properties						Freundlich			Linear		Initial antibiotic concentration range [3]	Solid/Liquid ratio	Reference
		pH	OC	CEC	Sand	Silt	Clay	K_f	n	Sub-dataset [1]	K_d	Sub-dataset [2]			
			%	mmol kg⁻¹	%	%	%	$mg^{1-1/n}$ $L^{1/n}$ kg^{-1}			L kg^{-1}		mg L^{-1}	g mL^{-1}	
1	China	6.1	1.5	175.0	38.0	46.5	15.5	3.34	1.29	A	1.73	B	1–40	1/2.5	[76]
2	Spain	5.0	3.1	129.3	58.0	22.0	20.0	3.40	1.10	A	2.50	B	0.71–14.24	1/7.5	[32]
3	Spain	5.1	3.0	98.5	64.0	17.0	19.0	4.80	1.40	A	1.70	B	0.71–14.24	1/7.5	[32]
4	Spain	4.3	3.4	59.2	58.0	19.0	22.0	3.80	1.10	A	2.60	B	0.71–14.24	1/7.5	[32]
5	Spain	4.5	2.8	53.1	61.0	18.0	20.0	4.20	1.20	A	2.70	B	0.71–14.24	1/7.5	[32]
6	Spain	4.4	2.4	58.9	62.0	20.0	17.0	4.60	1.10	a	3.40	b	0.71–14.24	1/7.5	[32]
7	Spain	4.0	1.7	52.1	53.0	27.0	20.0	3.10	1.10	a	2.60	b	0.71–14.24	1/7.5	[32]
8	Spain	3.8	2.0	47.4	60.0	19.0	21.0	5.60	1.10	A	4.40	B	0.71–14.24	1/7.5	[32]
9	Spain	4.7	3.9	65.9	51.0	21.0	29.0	5.60	1.10	A	4.20	B	0.71–14.24	1/7.5	[32]
10	Spain	4.9	3.8	64.8	65.0	17.0	19.0	7.30	1.20	A	4.50	B	0.71–14.24	1/7.5	[32]
11	Spain	4.4	3.7	51.3	62.0	20.0	19.0	4.90	1.10	A	4.10	b	0.71–14.24	1/7.5	[32]
12	Spain	4.2	2.4	42.5	65.0	15.0	21.0	5.10	1.10	a	3.70	b	0.71–14.24	1/7.5	[32]
13	Spain	4.1	1.2	44.7	64.0	19.0	17.0	3.00	1.10	a	2.00	b	0.71–14.24	1/7.5	[32]
14	Spain	4.2	2.5	46.5	66.0	16.0	18.0	5.90	1.20	A	3.60	b	0.71–14.24	1/7.5	[32]
15	Spain	3.9	1.7	52.1	43.0	27.0	30.0	3.70	1.20	A	1.90	B	0.71–14.24	1/7.5	[32]
16	Spain	4.4	1.3	38.8	66.0	15.0	18.0	3.10	1.20	A	1.60	B	0.71–14.24	1/7.5	[32]
17	Spain	4.1	1.3	40.0	68.0	14.0	17.0	3.10	1.20	A	1.70	B	0.71–14.24	1/7.5	[32]
18	Spain	3.8	2.7	63.6	54.0	24.0	21.0	6.00	1.30	a	3.00	B	0.71–14.24	1/7.5	[32]
19	Spain	3.7	1.7	44.4	59.0	22.0	20.0	4.10	1.10	a	3.00	b	0.71–14.24	1/7.5	[32]
20	Spain	4.3	1.1	40.8	70.0	12.0	18.0	2.00	1.10	A	1.40	b	0.71–14.24	1/7.5	[32]

21	Spain	4.0	1.6	47.4	55.0	22.0	23.0	3.50	1.10	A	2.40	b	0.71–14.24	1/7.5	[32]
22	Spain	4.0	1.9	46.1	58.0	23.0	19.0	3.40	1.20	A	2.10	B	0.71–14.24	1/7.5	[32]
23	Spain	4.4	2.1	52.9	61.0	16.0	23.0	3.50	1.10	A	2.70	B	0.71–14.24	1/7.5	[41]
24	Spain	4.1	2.1	52.5	55.0	26.0	19.0	4.40	1.20	a	2.80	B	0.71–14.24	1/7.5	[32]
25	Spain	3.8	1.6	41.5	57.0	25.0	18.0	4.30	1.20	A	2.50	B	0.71–14.24	1/7.5	[32]
26	Spain	4.0	2.0	43.0	64.0	19.0	17.0	3.50	1.00	A	3.00	B	0.71–14.24	1/7.5	[32]
27	Spain	4.2	1.1	42.4	69.0	13.0	18.0	2.50	1.10	A	1.70	B	0.71–14.24	1/7.5	[32]
28	Spain	4.5	1.1	42.5	67.0	14.0	19.0	2.80	1.20	a	1.50	B	0.71–14.24	1/7.5	[41]
29	Spain	4.5	5.3	116.5	41.0	26.0	34.0	11.20	1.00	A	11.10	b	0.71–14.24	1/7.5	[32]
30	Spain	4.2	3.3	65.1	57.0	20.0	23.0	6.50	1.20	A	4.40	b	0.71–14.24	1/7.5	[32]
31	Spain	6.2	1.7	164.7	56.0	20.0	24.0	0.60	1.20	A	0.30	b	0.71–14.24	1/7.5	[32]
32	Spain	3.7	5.0	76.9	51.0	22.0	27.0	NA [4]	NA	– [5]	15.10	B	0.71–14.24	1/7.5	[32]
33	Spain	4.1	1.8	44.9	48.0	23.0	29.0	4.30	1.20	A	2.70	B	0.71–14.24	1/7.5	[32]
34	Spain	4.5	1.3	43.2	63.0	17.0	19.0	2.70	1.20	A	1.50	B	0.71–14.24	1/7.5	[32]
35	Spain	4.7	2.6	71.8	53.0	22.0	26.0	5.60	1.20	A	3.50	B	0.71–14.24	1/7.5	[32]
36	Spain	4.2	1.4	56.2	58.0	22.0	20.0	3.90	1.20	a	2.50	B	0.71–14.24	1/7.5	[32]
37	Spain	5.0	2.4	61.9	66.0	17.0	18.0	5.00	1.20	A	2.70	B	0.71–14.24	1/7.5	[32]
38	Spain	4.3	1.8	49.5	62.0	22.0	16.0	5.10	1.20	A	3.40	B	0.71–14.24	1/7.5	[32]
39	Spain	4.3	2.5	63.9	59.0	19.0	22.0	5.80	1.20	A	3.60	B	0.71–14.24	1/7.5	[32]
40	Spain	4.2	2.2	54.5	63.0	20.0	17.0	4.80	1.20	A	2.80	b	0.71–14.24	1/7.5	[32]
41	Spain	4.2	2.3	43.9	65.0	18.0	17.0	5.40	1.20	A	3.10	b	0.71–14.24	1/7.5	[32]
42	Spain	4.4	2.5	54.0	62.0	20.0	18.0	5.10	1.20	A	3.10	B	0.71–14.24	1/7.5	[32]
43	Spain	4.6	3.8	68.2	48.0	27.0	25.0	5.80	1.20	A	3.60	B	0.71–14.24	1/7.5	[41]
44	Spain	4.1	3.1	53.3	65.0	14.0	21.0	3.70	1.00	a	3.70	B	0.71–14.24	1/7.5	[32]
45	Spain	4.5	3.7	67.3	63.0	18.0	19.0	6.30	1.20	A	3.60	B	0.71–14.24	1/7.5	[32]
46	Spain	4.1	2.7	50.3	48.0	31.0	21.0	5.70	1.20	A	3.50	b	0.71–14.24	1/7.5	[32]

47	Spain	4.2	3.3	54.5	49.0	29.0	22.0	8.00	1.20	A	5.10	b	0.71–14.24	1/7.5	[32]
48	Spain	4.5	3.5	48.2	59.0	21.0	20.0	6.60	1.30	A	3.30	b	0.71–14.24	1/7.5	[32]
49	Spain	4.5	3.7	60.4	61.0	20.0	19.0	4.50	1.00	a	4.30	B	0.71–14.24	1/7.5	[32]
50	Spain	4.4	2.8	87.8	55.0	22.0	23.0	5.80	1.20	A	3.80	B	0.71–14.24	1/7.5	[32]
51	Spain	4.0	10.9	116.4	49.0	19.0	32.0	NA	NA	–	23.10	B	0.71–14.24	1/7.5	[41]
52	Spain	6.4	4.6	303.1	44.0	39.0	18.0	6.70	1.20	A	3.60	B	0.71–14.24	1/7.5	[32]
53	Spain	5.9	2.0	149.6	22.0	62.0	16.0	1.90	1.20	a	1.00	B	0.71–14.24	1/7.5	[32]
54	Spain	4.5	3.0	67.6	39.0	47.0	14.0	6.70	1.10	A	5.00	B	0.71–14.24	1/7.5	[32]
55	Spain	5.5	2.8	110.9	35.0	45.0	20.0	5.70	1.10	A	4.10	B	0.71–14.24	1/7.5	[32]
56	Spain	6.2	2.9	234.1	26.0	51.0	23.0	6.40	1.20	A	3.50	B	0.71–14.24	1/7.5	[32]
57	Spain	5.9	6.1	263.0	30.0	44.0	25.0	13.70	1.30	A	7.90	B	0.71–14.24	1/7.5	[32]
58	Spain	5.2	1.5	67.5	30.0	55.0	15.0	4.80	1.30	A	2.30	B	0.71–14.24	1/7.5	[32]
59	Spain	5.7	2.3	128.6	26.0	58.0	16.0	5.00	1.20	A	2.60	B	0.71–14.24	1/7.5	[32]
60	Spain	4.3	4.3	60.4	41.0	42.0	17.0	10.80	1.20	a	7.30	B	0.71–14.24	1/7.5	[32]
61	Spain	4.1	1.4	102.6	48.0	25.0	26.0	4.50	1.10	A	3.20	B	0.71–14.24	1/7.5	[32]
62	Spain	6.4	1.8	125.4	27.0	56.0	17.0	1.20	1.30	A	0.60	B	0.71–14.24	1/7.5	[32]
63	Spain	5.4	2.0	144.5	25.0	60.0	15.0	4.50	1.20	A	2.80	B	0.71–14.24	1/7.5	[32]
64	Spain	5.0	3.6	86.2	25.0	59.0	17.0	8.90	1.30	A	5.10	B	0.71–14.24	1/7.5	[32]
65	Spain	5.5	1.6	80.3	30.0	47.0	23.0	4.10	1.20	A	2.20	B	0.71–14.24	1/7.5	[32]
66	Spain	5.4	6.9	173.8	40.0	42.0	18.0	NA	NA	–	11.70	B	0.71–14.24	1/7.5	[32]
67	Spain	5.4	7.8	151.5	40.0	42.0	19.0	15.10	1.20	A	10.30	B	0.71–14.24	1/7.5	[32]
68	Spain	6.2	8.3	335.3	45.0	39.0	16.0	7.70	1.30	A	NA	–	0.71–14.24	1/7.5	[32]
69	Spain	4.3	1.1	40.8	70.0	12.0	18.0	2.75	1.14	A	1.66	B	1.42–25.62	1/2.5	[32]
70	Spain	4.3	3.4	59.2	58.0	19.0	22.0	2.90	0.94	A	3.66	B	1.42–25.62	1/2.5	[32]
71	Spain	5.9	2.0	149.6	22.0	62.0	16.0	1.63	1.14	A	0.95	B	1.42–25.62	1/2.5	[32]
72	Spain	6.4	1.8	125.4	27.0	56.0	17.0	1.05	1.18	a	0.53	B	1.42–25.62	1/2.5	[13]

73	Spain	5.4	6.9	173.8	40.0	42.0	18.0	15.33	1.16	A	9.70	B	1.42–25.62	1/2.5	[13]
74	Canada	4.5	1.1	42.5	67.0	14.0	19.0	2.80	1.20	A	1.50	B	711.8–14236.0	1/2.5	[36]
75	Canada	4.4	2.1	52.9	61.0	16.0	23.0	3.50	1.10	A	2.70	B	711.8–14236.0	1/2.5	[36]
76	Canada	4.6	3.8	68.2	48.0	27.0	25.0	5.80	1.20	a	3.60	B	711.8–14236.0	1/2.5	[36]
77	Canada	4.5	5.3	116.5	41.0	26.0	34.0	11.20	1.00	A	11.10	b	711.8–14236.0	1/2.5	[36]
78	Canada	4.0	10.9	116.4	49.0	19.0	32.0	NA	NA	–	23.10	B	711.8–14236.0	1/2.5	[36]
79	UK	6.8	3.1	224.0	42.6	32.3	25.1	2.50	1.08	A	16.60	B	1.50	1/2	[54]
80	UK	6.6	2.2	114.0	69.2	20.5	10.3	1.50	1.05	A	8.10	B	1.50	1/2	[54]
81	Brazil	3.7	1.2	35.7	77.9	4.0	18.1	NA	NA	–	1.60	B	1.00	1/2	[14]
82	Brazil	6.9	9.6	52.2	10.9	20.7	68.4	NA	NA	–	6.10	B	1.00	1/2	[14]
83	Brazil	5.1	0.7	32.0	84.0	10.0	6.0	NA	NA	–	0.70	B	1.00	1/2	[14]
84	Brazil	5.7	4.1	217.5	18.6	44.8	36.6	NA	NA	–	5.30	B	1.00	1/2	[14]
85	Brazil	3.8	1.0	27.6	88.0	4.0	8.0	NA	NA	–	1.10	B	1.00	1/2	[14]
86	Brazil	4.8	2.6	153.2	51.2	34.6	14.2	NA	NA	–	7.00	B	1.00	1/2	[14]
87	Brazil	5.1	5.4	125.2	47.3	18.2	34.5	NA	NA	–	8.20	B	1.00	1/2	[14]
88	Brazil	3.7	2.9	56.9	65.6	10.1	24.3	NA	NA	–	4.60	B	1.00	1/2	[14]
89	Brazil	4.8	2.2	100.0	44.9	34.7	20.4	NA	NA	–	6.70	B	1.00	1/2	[14]
90	Brazil	4.5	1.0	39.2	72.0	24.0	4.0	NA	NA	–	1.30	B	1.00	1/2	[14]
91	Brazil	5.4	5.8	207.8	20.6	25.1	54.3	NA	NA	–	13.30	B	1.00	1/2	[14]
92	New Zealand	6.1	2.1	154.0	11.0	27.0	62.0	NA	NA	–	1.87	B	1.5–15	1/15	[12]
93	New Zealand	6.7	5.0	223.0	9.0	37.0	54.0	8.01	1.25	A	5.07	B	1.5–15	1/15	[12]
94	New Zealand	5.8	4.0	215.0	19.0	30.0	51.0	14.49	1.20	A	9.53	B	1.5–15	1/15	[12]
95	New Zealand	5.7	8.2	356.0	34.0	17.0	48.0	10.98	1.08	A	10.59	B	1.5–15	1/15	[12]
96	New Zealand	5.1	3.3	97.0	87.0	2.0	11.0	11.47	1.18	A	8.60	B	1.5–15	1/15	[12]
97	New Zealand	6.9	1.1	76.0	45.0	14.0	41.0	NA	NA	–	8.76	B	1.5–15	1/15	[12]
98	New Zealand	6.1	2.1	154.0	11.0	62.0	27.0	0.97	0.93	A	1.00	B	1.5–15	1/15	[58]

99	New Zealand	5.8	4.0	215.0	19.0	51.0	30.0	2.52	0.90	A	2.63	B	1.5–15	1/15	[58]
100	China	6.5	1.2	246.6	30.5	34.8	34.8	2.12	2.98	A	0.32	B	1–25	1/4	[77]

[1] Sub-datasets "A" and "a" were used for development and validation of the models for estimating K_f, respectively.

[2] Sub-datasets "B" and "b" were used for development and validation of the models for estimating K_d, respectively.

[3] $CaCl_2$ was used as background electrolyte in all batch sorption tests.

[4] "NA" means that the value was not reported in the cited reference.

[5] "–" denotes an exclusion from any sub-datasets.

Table S3. SMT sorption parameters and associated soil properties.

No.	Origin	Soil properties						Freundlich			Linear		Initial antibiotic concentration range [3]	Solid/Liquid ratio	Reference
		pH	OC	CEC	Sand	Silt	Clay	K_f	n	Sub-dataset [1]	K_d	Sub-dataset [2]			
			%	mmol kg^{-1}	%	%	%	mg$^{1-1/n}$ L$^{1/n}$ kg^{-1}			L kg^{-1}		mg L^{-1}	g mL^{-1}	
1	USA	7.1	2.2	233.0	NA [4]	NA	22.3	3.02	1.35	a	1.86	b	0–13.917	1/2.5	[33]
2	USA	7.0	3.0	268.0	NA	NA	24.5	4.27	1.27	a	3.03	b	0–13.917	1/2.5	[33]
3	USA	6.7	2.3	254.0	NA	NA	25.3	4.07	1.52	a	2.21	b	0–13.917	1/2.5	[33]
4	USA	6.6	1.3	138.0	NA	NA	19.9	2.75	1.59	a	1.32	b	0–13.917	1/2.5	[33]
5	USA	5.2	1.2	90.0	NA	NA	15.8	NA	NA	– [5]	1.69	b	0–13.917	1/2.5	[33]
6	USA	4.7	2.3	151.0	NA	NA	19.0	NA	NA	–	4.98	b	0–13.917	1/2.5	[33]
7	USA	6.3	1.9	180.0	NA	NA	21.1	3.63	1.49	a	1.97	b	0–13.917	1/2.5	[33]
8	USA	6.2	2.0	226.0	NA	NA	24.7	3.89	1.47	a	2.18	b	0–13.917	1/2.5	[33]
9	USA	5.8	2.2	235.0	NA	NA	24.7	4.68	1.45	a	2.77	b	0–13.917	1/2.5	[33]
10	Spain	4.5	1.1	42.5	67.0	14.0	19.0	1.30	1.10	A	0.80	B	695.75–13915	1/2.5	[41]
11	Spain	4.4	2.1	52.9	61.0	16.0	23.0	2.00	1.20	A	1.10	B	695.75–13915	1/2.5	[41]
12	Spain	4.6	3.8	68.2	48.0	27.0	25.0	3.40	1.20	A	1.70	b	695.75–13915	1/2.5	[41]
13	Spain	4.5	5.3	116.5	41.0	26.0	34.0	5.50	1.10	a	4.40	B	695.75–13915	1/2.5	[41]
14	Spain	4.0	10.9	116.4	49.0	19.0	32.0	16.00	1.10	a	14.20	B	695.75–13915	1/2.5	[41]
15	Spain	5.0	3.1	58.0	58.0	22.0	20.0	2.10	1.20	A	1.00	B	0.69585–13.917	1/7.5	[32]
16	Spain	5.1	3.0	64.0	64.0	17.0	19.0	1.60	1.20	A	0.90	B	0.69585–13.917	1/7.5	[32]
17	Spain	4.3	3.4	58.0	58.0	19.0	22.0	1.30	1.10	A	1.00	B	0.69585–13.917	1/7.5	[32]
18	Spain	4.5	2.8	61.0	61.0	18.0	20.0	1.80	1.10	A	1.20	B	0.69585–13.917	1/7.5	[32]
19	Spain	4.4	2.4	62.0	62.0	20.0	17.0	2.30	1.10	A	1.60	b	0.69585–13.917	1/7.5	[32]

20	Spain	4.0	1.7	53.0	53.0	27.0	20.0	2.20	1.20	a	1.10	b	0.69585–13.917	1/7.5	[32]
21	Spain	3.8	2.0	60.0	60.0	19.0	21.0	2.60	1.10	a	2.00	b	0.69585–13.917	1/7.5	[32]
22	Spain	4.7	3.9	51.0	51.0	21.0	29.0	2.30	1.10	a	1.70	B	0.69585–13.917	1/7.5	[32]
23	Spain	4.9	3.8	65.0	65.0	17.0	19.0	2.80	1.10	A	2.00	B	0.69585–13.917	1/7.5	[32]
24	Spain	4.4	3.7	62.0	62.0	20.0	19.0	1.90	1.00	A	1.80	B	0.69585–13.917	1/7.5	[32]
25	Spain	4.2	2.4	65.0	65.0	15.0	21.0	2.32	1.10	A	1.60	B	0.69585–13.917	1/7.5	[32]
26	Spain	4.1	1.2	64.0	64.0	19.0	17.0	1.30	1.20	A	0.80	B	0.69585–13.917	1/7.5	[32]
27	Spain	4.2	2.5	66.0	66.0	16.0	18.0	1.90	1.10	A	1.30	B	0.69585–13.917	1/7.5	[32]
28	Spain	3.9	1.7	43.0	43.0	27.0	30.0	2.50	1.30	A	0.90	B	0.69585–13.917	1/7.5	[32]
29	Spain	4.4	1.3	66.0	66.0	15.0	18.0	2.00	1.30	A	0.80	B	0.69585–13.917	1/7.5	[32]
30	Spain	4.1	1.3	68.0	68.0	14.0	17.0	1.40	1.10	a	0.90	B	0.69585–13.917	1/7.5	[32]
31	Spain	3.8	2.7	54.0	54.0	24.0	21.0	2.50	1.10	a	1.80	B	0.69585–13.917	1/7.5	[32]
32	Spain	3.7	1.7	59.0	59.0	22.0	20.0	2.90	1.20	a	1.50	b	0.69585–13.917	1/7.5	[32]
33	Spain	4.3	1.1	70.0	70.0	12.0	18.0	0.90	1.10	A	0.70	b	0.69585–13.917	1/7.5	[32]
34	Spain	4.0	1.6	55.0	55.0	22.0	23.0	2.20	1.20	A	1.20	b	0.69585–13.917	1/7.5	[32]
35	Spain	4.0	1.9	58.0	58.0	23.0	19.0	1.70	1.20	A	1.00	B	0.69585–13.917	1/7.5	[32]
36	Spain	4.4	2.1	61.0	61.0	16.0	23.0	2.00	1.20	A	1.10	B	0.69585–13.917	1/7.5	[32]
37	Spain	4.1	2.1	55.0	55.0	26.0	19.0	2.60	1.20	A	1.40	B	0.69585–13.917	1/7.5	[32]
38	Spain	3.8	1.6	57.0	57.0	25.0	18.0	2.00	1.10	A	1.30	B	0.69585–13.917	1/7.5	[32]
39	Spain	4.0	2.0	64.0	64.0	19.0	17.0	1.10	1.00	A	1.30	B	0.69585–13.917	1/7.5	[32]
40	Spain	4.2	1.1	69.0	69.0	13.0	18.0	1.40	1.10	A	0.90	B	0.69585–13.917	1/7.5	[32]
41	Spain	4.5	1.1	67.0	67.0	14.0	19.0	1.30	1.10	a	0.80	B	0.69585–13.917	1/7.5	[32]
42	Spain	4.5	5.3	41.0	41.0	26.0	34.0	5.50	1.10	A	4.40	B	0.69585–13.917	1/7.5	[32]
43	Spain	4.2	3.3	57.0	57.0	20.0	23.0	2.30	1.10	a	1.60	B	0.69585–13.917	1/7.5	[32]
44	Spain	6.2	1.7	56.0	56.0	20.0	24.0	1.30	1.30	A	0.50	b	0.69585–13.917	1/7.5	[32]
45	Spain	3.7	5.0	51.0	51.0	22.0	27.0	6.90	1.10	A	5.70	b	0.69585–13.917	1/7.5	[32]

46	Spain	4.1	1.8	48.0	48.0	23.0	29.0	2.30	1.30	A	1.00	b	0.69585–13.917	1/7.5	[32]
47	Spain	4.5	1.3	63.0	63.0	17.0	19.0	1.30	1.20	A	0.60	B	0.69585–13.917	1/7.5	[32]
48	Spain	4.7	2.6	53.0	53.0	22.0	26.0	3.30	1.30	A	1.50	B	0.69585–13.917	1/7.5	[32]
49	Spain	4.2	1.4	58.0	58.0	22.0	20.0	2.40	1.30	A	1.10	B	0.69585–13.917	1/7.5	[32]
50	Spain	5.0	2.4	66.0	66.0	17.0	18.0	3.00	1.20	a	1.50	B	0.69585–13.917	1/7.5	[32]
51	Spain	4.3	1.8	62.0	62.0	22.0	16.0	3.40	1.30	a	1.40	B	0.69585–13.917	1/7.5	[32]
52	Spain	4.3	2.5	59.0	59.0	19.0	22.0	3.20	1.30	a	1.50	B	0.69585–13.917	1/7.5	[32]
53	Spain	4.2	2.2	63.0	63.0	20.0	17.0	2.50	1.30	A	1.10	B	0.69585–13.917	1/7.5	[32]
54	Spain	4.2	2.3	65.0	65.0	18.0	17.0	2.90	1.30	A	1.20	b	0.69585–13.917	1/7.5	[32]
55	Spain	4.4	2.5	62.0	62.0	20.0	18.0	2.30	1.20	A	1.20	b	0.69585–13.917	1/7.5	[32]
56	Spain	4.6	3.8	48.0	48.0	27.0	25.0	3.40	1.20	A	1.70	b	0.69585–13.917	1/7.5	[32]
57	Spain	4.1	3.1	65.0	65.0	14.0	21.0	2.30	1.10	A	1.50	B	0.69585–13.917	1/7.5	[32]
58	Spain	4.5	3.7	63.0	63.0	18.0	19.0	2.70	1.20	a	1.40	B	0.69585–13.917	1/7.5	[32]
59	Spain	4.1	2.7	48.0	48.0	31.0	21.0	3.80	1.30	a	1.60	B	0.69585–13.917	1/7.5	[32]
60	Spain	4.2	3.3	49.0	49.0	29.0	22.0	3.20	1.10	A	2.20	B	0.69585–13.917	1/7.5	[32]
61	Spain	4.5	3.5	59.0	59.0	21.0	20.0	3.90	1.30	A	1.60	B	0.69585–13.917	1/7.5	[32]
62	Spain	4.5	3.7	61.0	61.0	20.0	19.0	3.20	1.20	A	1.90	B	0.69585–13.917	1/7.5	[32]
63	Spain	4.4	2.8	55.0	55.0	22.0	23.0	2.60	1.20	A	1.30	B	0.69585–13.917	1/7.5	[32]
64	Spain	4.0	10.9	49.0	49.0	19.0	32.0	16.00	1.10	a	14.20	b	0.69585–13.917	1/7.5	[32]
65	Spain	6.4	4.6	44.0	44.0	39.0	18.0	NA	NA	−	3.90	B	0.69585–13.917	1/7.5	[32]
66	Spain	6.2	8.3	45.0	45.0	39.0	16.0	8.00	1.20	A	4.50	B	0.69585–13.917	1/7.5	[32]
67	Spain	5.9	2.0	22.0	22.0	62.0	16.0	2.30	1.40	A	0.70	B	0.69585–13.917	1/7.5	[32]
68	Spain	4.5	3.0	39.0	39.0	47.0	14.0	3.50	1.20	A	1.90	B	0.69585–13.917	1/7.5	[32]
69	Spain	5.5	2.8	35.0	35.0	45.0	20.0	5.00	1.30	A	2.10	B	0.69585–13.917	1/7.5	[32]
70	Spain	6.2	2.9	26.0	26.0	51.0	23.0	6.10	1.40	A	2.50	B	0.69585–13.917	1/7.5	[32]
71	Spain	5.1	4.6	37.0	37.0	45.0	19.0	NA	NA	−	9.20	b	0.69585–13.917	1/7.5	[32]

72	Spain	5.9	6.1	30.0	30.0	44.0	25.0	NA	NA	–	9.20	B	0.69585–13.917	1/7.5	[32]
73	Spain	5.2	1.5	30.0	30.0	55.0	15.0	3.30	1.40	A	1.10	B	0.69585–13.917	1/7.5	[32]
74	Spain	5.7	2.3	26.0	26.0	58.0	16.0	4.50	1.50	A	1.40	B	0.69585–13.917	1/7.5	[32]
75	Spain	4.3	4.3	41.0	41.0	42.0	17.0	4.80	1.30	A	2.30	B	0.69585–13.917	1/7.5	[32]
76	Spain	4.1	1.4	48.0	48.0	25.0	26.0	3.10	1.30	A	1.30	B	0.69585–13.917	1/7.5	[32]
77	Spain	6.4	1.8	27.0	27.0	56.0	17.0	2.90	1.40	A	1.10	B	0.69585–13.917	1/7.5	[32]
78	Spain	5.4	2.0	25.0	25.0	60.0	15.0	2.70	1.20	A	1.50	B	0.69585–13.917	1/7.5	[32]
79	Spain	5.0	3.6	25.0	25.0	59.0	17.0	3.40	1.10	A	2.30	B	0.69585–13.917	1/7.5	[32]
80	Spain	5.5	1.6	30.0	30.0	47.0	23.0	3.00	1.40	A	1.10	B	0.69585–13.917	1/7.5	[32]
81	Spain	5.4	6.9	40.0	40.0	42.0	18.0	8.00	1.20	A	5.00	B	0.69585–13.917	1/7.5	[32]
82	Spain	5.4	7.8	40.0	40.0	42.0	19.0	8.60	1.20	A	5.80	B	0.69585–13.917	1/7.5	[32]
83	Spain	4.3	1.1	40.8	70.0	12.0	18.0	1.67	1.30	A	0.57	B	1.3915–25.047	1/2.5	[5]
84	Spain	4.3	3.4	59.2	58.0	19.0	22.0	1.24	1.00	A	1.24	B	1.3915–25.047	1/2.5	[5]
85	Spain	4.0	10.9	116.4	49.0	19.0	32.0	15.79	1.11	a	11.30	B	1.3915–25.047	1/2.5	[5]
86	Spain	5.9	2.0	149.6	22.0	62.0	16.0	1.94	1.28	A	0.70	b	1.3915–25.047	1/2.5	[5]
87	Spain	6.4	1.8	125.4	27.0	56.0	17.0	2.40	1.37	A	0.67	B	1.3915–25.047	1/2.5	[5]
88	Spain	5.4	6.9	173.8	40.0	42.0	18.0	7.48	1.19	A	3.98	B	1.3915–25.047	1/2.5	[5]
89	Brazil	5.0	0.9	19.3	91.1	1.8	6.2	0.90	1.32	A	0.36	B	1–75	1/1	[53]
90	Brazil	4.9	1.7	52.7	14.9	30.2	54.6	3.30	1.25	A	1.78	B	1–75	1/1	[53]
91	Brazil	4.1	1.4	51.9	52.9	10.5	36.2	3.70	1.15	a	2.56	b	1–75	1/1	[53]
92	Brazil	4.4	1.9	66.0	43.5	7.0	49.2	2.60	1.20	A	1.51	B	1–75	1/1	[53]
93	Croatia	5.8	1.8	44.8	99.7	0.3	0.1	4.25	0.92	A	4.46	B	3–50	1/2	[80]
94	Croatia	7.3	0.9	15.8	99.9	0.1	0.1	1.48	0.96	A	1.45	B	3–50	1/2	[80]
95	Croatia	6.5	7.4	70.0	100.0	0.0	0.0	7.99	0.85	A	8.10	B	3–50	1/5	[80]
96	Croatia	7.1	2.0	51.1	99.8	0.2	0.1	4.40	1.13	A	4.66	B	3–50	1/2	[80]
97	Croatia	6.4	4.3	86.5	99.7	0.3	0.1	7.69	1.06	a	7.93	B	3–50	1/2	[80]

98	Croatia	7.2	1.2	91.0	99.6	0.3	0.1	3.04	1.12	A	2.99	b	3–50	1/2	[80]
99	Croatia	6.6	2.9	31.4	99.9	0.3	0.1	5.76	1.15	A	5.76	b	3–50	1/2	[80]
100	Croatia	6.5	1.2	34.5	99.7	0.3	0.1	2.82	1.07	a	2.48	B	3–50	1/2	[80]
101	Croatia	5.8	1.8	44.8	99.7	0.3	0.1	1.16	0.84	A	4.45	B	3–50	1/2	[80]
102	Croatia	7.3	0.9	15.8	99.9	0.1	0.1	1.21	0.77	A	1.62	B	3–50	1/2	[80]
103	Croatia	6.5	7.4	70.0	100.0	0.0	0.0	7.16	0.78	A	7.29	B	3–50	1/5	[80]
104	Croatia	7.1	2.0	51.1	99.8	0.2	0.1	2.08	0.87	A	2.17	B	3–50	1/2	[80]
105	Croatia	6.4	4.3	86.5	99.7	0.3	0.1	7.70	0.78	a	7.79	B	3–50	1/2	[80]
106	Croatia	7.2	1.2	91.0	99.6	0.3	0.1	1.63	1.13	A	1.31	B	3–50	1/2	[80]
107	Croatia	6.6	2.9	31.4	99.9	0.3	0.1	5.11	0.90	A	5.26	b	3–50	1/2	[80]
108	Croatia	6.5	1.2	34.5	99.7	0.3	0.1	2.60	1.07	a	2.41	b	3–50	1/2	[80]
109	Croatia	5.8	1.8	44.8	99.7	0.3	0.1	2.14	1.15	A	1.91	b	3–50	1/2	[80]
110	Croatia	7.3	0.9	15.8	99.9	0.1	0.1	1.79	1.16	A	0.45	B	3–50	1/2	[80]
111	Croatia	6.5	7.4	70.0	100.0	0.0	0.0	3.51	0.99	a	3.31	B	3–50	1/5	[80]
112	Croatia	7.1	2.0	51.1	99.8	0.2	0.1	1.31	1.41	A	0.48	B	3–50	1/2	[80]
113	Croatia	6.4	4.3	86.5	99.7	0.3	0.1	2.13	1.21	A	1.71	B	3–50	1/2	[80]
114	Croatia	7.2	1.2	91.0	99.6	0.3	0.1	1.29	1.66	A	0.44	B	3–50	1/2	[80]
115	Croatia	6.6	2.9	31.4	99.9	0.3	0.1	3.99	1.18	A	1.83	B	3–50	1/2	[80]
116	Croatia	6.5	1.2	34.5	99.7	0.3	0.1	1.41	1.38	A	0.25	B	3–50	1/2	[80]
117	China	6.9	0.9	108.4	11.6	36.3	52.0	NA	NA	−	2.90	B	0.1–9.8	1/20	[29]
118	China	5.5	0.5	148.7	41.0	35.8	23.3	2.94	1.27	A	1.97	B	0.1–9.8	1/20 [2]	[29]
119	China	5.0	1.0	88.7	25.1	28.9	46.0	NA	NA	−	5.45	B	0.1–9.8	1/20	[29]
120	China	5.1	4.3	276.0	40.5	45.5	14.0	6.55	1.25	A	1.95	B	1– 50	1/10	[79]
121	China	7.3	1.0	106.0	43.5	21.0	35.5	4.64	0.82	A	NA	−	1– 50	1/10	[79]
122	China	5.1	4.3	276.0	40.5	45.5	14.0	6.55	1.25	A	3.31	B	1–50	1/10	[35]
123	China	7.3	1.0	106.0	43.5	21.0	35.5	4.64	0.82	A	NA	−	1–50	1/10	[35]

124	Korea	6.0	0.4	20.7	84.1	6.2	9.7	NA	NA	–	3.67	B	2.5–50	1/20	[81]
125	Korea	6.4	2.9	23.4	64.1	26.1	9.8	2.64	0.41	A	2.24	B	2.5-–50	1/20	[81]
126	Korea	5.7	2.2	45.1	74.5	20.0	5.5	1.19	1.41	A	0.95	B	5–10	1/5	[20]
127	Korea	5.5	4.0	60.5	65.2	26.7	8.1	NA	NA	–	0.98	B	5–10	1/5	[20]
128	Italy	7.8	2.8	NA	72.7	10.6	16.6	2.06	1.10	a	1.65	b	6.7–26.7	1/2	[82]
129	Italy	5.3	1.7	NA	81.7	6.0	12.3	1.20	1.09	a	0.97	b	6.7–26.7	1/2	[82]
130	China	7.5	0.4	60.0	36.0	40.0	24.0	0.87	4.00	A	0.11	B	0.5–20	1/10	[83]
131	New Zealand	6.1	2.1	154.0	11.0	62.0	27.0	1.10	1.01	A	1.07	B	1.5–15	1/15	[58]
132	New Zealand	5.8	4.0	215.0	19.0	51.0	30.0	2.19	1.09	A	1.82	B	1.5–15	1/15	[58]
133	New Zealand	6.1	2.1	154.0	11.0	27.0	62.0	4.94	2.00	A	1.66	B	1.5–15	1/15	[12]
134	New Zealand	6.7	5.0	223.0	9.0	37.0	54.0	5.15	1.45	A	2.61	B	1.5–15	1/15	[12]
135	New Zealand	5.8	4.0	215.0	19.0	30.0	51.0	5.34	1.35	a	2.92	B	1.5–15	1/15	[12]
136	New Zealand	5.7	8.2	356.0	34.0	17.0	48.0	5.98	1.04	A	5.08	b	1.5–15	1/15	[12]
137	New Zealand	5.1	3.3	97.0	87.0	2.0	11.0	2.00	1.47	A	0.96	B	1.5–15	1/15	[12]
138	New Zealand	6.9	1.1	76.0	45.0	14.0	41.0	0.83	0.93	A	1.45	B	1.5–15	1/15	[12]
139	USA	5.3	1.0	68.0	77.0	18.0	5.2	3.60	1.24	A	4.60	B	0.25–4.0	1/6	[72]
140	USA	4.9	1.1	130.0	46.0	38.0	16.0	2.80	1.47	A	4.50	B	0.25–4.0	1/6	[72]
141	China	4.0	1.0	96.9	16.9	67.2	15.9	1.39	1.01	A	1.39	B	0.05–1	1/25	[84]
142	China	5.8	6.1	312.0	11.3	73.1	15.6	3.97	1.09	A	4.08	B	0.05–1	1/25	[84]
143	China	6.2	5.0	180.0	4.8	75.8	19.4	4.39	1.02	a	4.42	b	0.05–1	1/25	[84]
144	China	7.6	2.4	106.0	16.5	74.3	9.2	1.19	1.33	A	1.27	B	0.05–1	1/25	[84]
145	China	5.6	1.0	218.0	2.4	82.9	14.7	0.87	1.09	A	0.89	B	0.05–1	1/25	[84]
146	China	5.7	0.2	237.0	22.0	51.0	27.0	NA	NA	A	3.81	B	1–10	1/4	[85]
147	USA	8.2	0.1	121.0	56.0	26.0	18.0	0.13	1.32	A	0.32	B	1.1–22.2	1/3	[56]
148	USA	7.8	1.4	107.0	68.0	22.0	10.0	0.35	1.17	A	0.55	B	1.1–22.2	1/3	[56]
149	USA	5.4	2.2	157.0	46.0	34.0	20.0	4.77	0.72	a	2.52	b	1.1–22.2	1/3	[56]

150	USA	5.5	2.7	200.0	44.0	38.0	18.0	3.98	0.97	A	3.77	B	1.1–22.2	1/3	[56]
151	USA	8.2	3.8	233.0	38.0	36.0	26.0	1.77	0.98	A	1.70	B	1.1–22.2	1/3	[56]
152	USA	7.9	1.9	NA	15.0	51.0	34.0	NA	NA	–	1.25	b	0.012–1.219	1/5	[78]
153	USA	7.8	2.5	NA	12.0	62.0	26.0	NA	NA	–	1.46	b	0.012–1.219	1/5	[78]
154	USA	7.9	2.4	NA	46.0	34.0	20.0	NA	NA	–	1.99	b	0.012–1.219	1/5	[78]
155	China	8.6	0.2	212.1	36.0	40.0	24.0	0.34	0.93	A	0.42	B	1.0–25	1/10	[86]

[1] Sub-datasets "A" and "a" were used for development and validation of the models for estimating K_f, respectively.

[2] Sub-datasets "B" and "b" were used for development and validation of the models for estimating K_d, respectively.

[3] $CaCl_2$ was used as background electrolyte in all batch sorption tests.

[4] "NA" means that the value was not reported in the cited reference.

[5] "–" denotes an exclusion from any sub-datasets.

Table S4. SDZ sorption parameters and associated soil properties.

No.	Origin	Soil properties						Freundlich			Linear		Initial antibiotic concentration range [3]	Solid/Liquid ratio	Reference
		pH	OC	CEC	Sand	Silt	Clay	K_f	n	Sub-dataset [1]	K_d	Sub-dataset [2]			
			%	mmol kg⁻¹	%	%	%	$mg^{1-1/n}$ $L^{1/n}$ kg^{-1}			L kg^{-1}		mg L^{-1}	g mL^{-1}	
1	Spain	4.5	1.1	42.5	67.0	14.0	19.0	1.00	1.00	A	0.90	B	625.75–12515	1/2.5	[41]
2	Spain	4.4	2.1	52.9	61.0	16.0	23.0	1.70	1.10	A	1.30	B	625.75–12515	1/2.5	[41]
3	Spain	4.6	3.8	68.2	48.0	27.0	25.0	2.40	1.10	A	1.90	B	625.75–12515	1/2.5	[41]
4	Spain	4.5	5.3	116.5	41.0	26.0	34.0	4.30	1.00	a	4.50	B	625.75–12515	1/2.5	[41]
5	Spain	4.0	10.9	116.4	49.0	19.0	32.0	NA [4]	NA	– [5]	12.00	b	625.75–12515	1/2.5	[41]
6	Spain	5.0	3.1	129.3	58.0	22.0	20.0	1.39	0.91	A	1.20	B	0.625–12.5	1/2.5	[31]
7	Spain	5.1	3.0	98.5	64.0	17.0	19.0	1.21	0.83	A	0.80	B	0.625–12.5	1/2.5	[31]
8	Spain	4.3	3.4	59.2	58.0	19.0	22.0	1.57	0.91	B	1.30	B	0.625–12.5	1/2.5	[31]
9	Spain	4.5	2.8	53.1	61.0	18.0	20.0	1.03	1.11	A	1.20	B	0.625–12.5	1/2.5	[31]
10	Spain	4.4	2.4	58.9	62.0	20.0	17.0	1.30	1.00	a	1.30	B	0.625–12.5	1/2.5	[31]
11	Spain	4.0	1.7	52.1	53.0	27.0	20.0	1.59	0.83	a	1.00	b	0.625–12.5	1/2.5	[31]
12	Spain	3.8	2.0	47.4	60.0	19.0	21.0	1.38	1.11	a	1.70	b	0.625–12.5	1/2.5	[31]
13	Spain	4.7	3.9	65.9	51.0	21.0	29.0	1.61	1.11	A	1.90	B	0.625–12.5	1/2.5	[31]
14	Spain	4.9	3.8	64.8	65.0	17.0	19.0	2.10	1.00	A	1.90	B	0.625–12.5	1/2.5	[31]
15	Spain	4.4	3.7	51.3	62.0	20.0	19.0	1.39	0.91	A	1.30	B	0.625–12.5	1/2.5	[31]
16	Spain	4.2	2.4	42.5	65.0	15.0	21.0	1.57	0.91	A	1.40	B	0.625–12.5	1/2.5	[31]
17	Spain	4.1	1.2	44.7	64.0	19.0	17.0	1.14	0.83	A	0.70	B	0.625–12.5	1/2.5	[31]
18	Spain	4.2	2.5	46.5	66.0	16.0	18.0	1.50	1.00	A	1.20	B	0.625–12.5	1/2.5	[31]
19	Spain	3.9	1.7	52.1	43.0	27.0	30.0	1.32	0.77	A	1.00	B	0.625–12.5	1/2.5	[31]
20	Spain	4.4	1.3	38.8	66.0	15.0	18.0	1.13	0.91	A	0.80	B	0.625–12.5	1/2.5	[31]

21	Spain	4.1	1.3	40.0	68.0	14.0	17.0	0.87	0.91	A	0.70	B	0.625–12.5	1/2.5	[31]
22	Spain	3.8	2.7	63.6	54.0	24.0	21.0	2.27	0.83	A	1.50	b	0.625–12.5	1/2.5	[31]
23	Spain	3.7	1.7	44.4	59.0	22.0	20.0	3.03	0.83	a	2.20	B	0.625–12.5	1/2.5	[31]
24	Spain	4.3	1.1	40.8	70.0	12.0	18.0	0.46	1.11	a	0.60	B	0.625–12.5	1/2.5	[31]
25	Spain	4.0	1.6	47.4	55.0	22.0	23.0	1.20	1.00	a	1.00	B	0.625–12.5	1/2.5	[31]
26	Spain	4.0	1.9	46.1	58.0	23.0	19.0	1.59	0.83	A	1.10	B	0.625–12.5	1/2.5	[31]
27	Spain	4.4	2.1	52.9	61.0	16.0	23.0	1.48	0.91	A	1.30	b	0.625–12.5	1/2.5	[31]
28	Spain	4.1	2.1	52.5	55.0	26.0	19.0	1.40	1.00	A	1.30	B	0.625–12.5	1/2.5	[31]
29	Spain	3.8	1.6	41.5	57.0	25.0	18.0	1.00	1.00	A	1.10	B	0.625–12.5	1/2.5	[31]
30	Spain	4.0	2.0	43.0	64.0	19.0	17.0	1.20	1.00	A	1.20	B	0.625–12.5	1/2.5	[31]
31	Spain	4.2	1.1	42.4	69.0	13.0	18.0	0.57	1.11	A	0.70	B	0.625–12.5	1/2.5	[31]
32	Spain	4.5	1.1	42.5	67.0	14.0	19.0	1.00	1.00	A	0.90	B	0.625–12.5	1/2.5	[31]
33	Spain	4.5	5.3	116.5	41.0	26.0	34.0	4.30	1.00	a	4.50	B	0.625–12.5	1/2.5	[31]
34	Spain	4.2	3.3	65.1	57.0	20.0	23.0	1.70	1.00	A	1.50	B	0.625–12.5	1/2.5	[31]
35	Spain	6.2	1.7	164.7	56.0	20.0	24.0	0.55	0.67	A	0.30	B	0.625–12.5	1/2.5	[31]
36	Spain	3.7	5.0	76.9	51.0	22.0	27.0	NA	NA	–	5.90	b	0.625–12.5	1/2.5	[31]
37	Spain	4.1	1.8	44.9	48.0	23.0	29.0	1.29	0.83	A	0.90	B	0.625–12.5	1/2.5	[31]
38	Spain	4.5	1.3	43.2	63.0	17.0	19.0	0.78	0.91	A	0.60	B	0.625–12.5	1/2.5	[31]
39	Spain	4.7	2.6	71.8	53.0	22.0	26.0	1.39	0.91	A	1.20	B	0.625–12.5	1/2.5	[31]
40	Spain	4.2	1.4	56.2	58.0	22.0	20.0	1.00	1.00	A	1.10	B	0.625–12.5	1/2.5	[31]
41	Spain	5.0	2.4	61.9	66.0	17.0	18.0	2.24	0.77	a	1.50	B	0.625–12.5	1/2.5	[31]
42	Spain	4.3	1.8	49.5	62.0	22.0	16.0	1.65	0.91	a	1.20	B	0.625–12.5	1/2.5	[31]
43	Spain	4.3	2.5	63.9	59.0	19.0	22.0	1.57	0.91	a	1.30	B	0.625–12.5	1/2.5	[31]
44	Spain	4.2	2.2	54.5	63.0	20.0	17.0	1.10	1.00	a	1.10	b	0.625–12.5	1/2.5	[31]
45	Spain	4.2	2.3	43.9	65.0	18.0	17.0	1.31	0.91	A	1.10	b	0.625–12.5	1/2.5	[31]
46	Spain	4.4	2.5	54.0	62.0	20.0	18.0	1.22	0.91	A	1.10	B	0.625–12.5	1/2.5	[31]

47	Spain	4.6	3.8	68.2	48.0	27.0	25.0	2.09	0.91	A	1.90	B	0.625–12.5	1/2.5	[31]
48	Spain	4.1	3.1	53.3	65.0	14.0	21.0	1.83	0.91	A	1.70	B	0.625–12.5	1/2.5	[31]
49	Spain	4.5	3.7	67.3	63.0	18.0	19.0	1.90	1.00	a	1.60	B	0.625–12.5	1/2.5	[31]
50	Spain	4.1	2.7	50.3	48.0	31.0	21.0	2.20	0.83	A	1.60	B	0.625–12.5	1/2.5	[31]
51	Spain	4.2	3.3	54.5	49.0	29.0	22.0	3.03	0.83	a	2.50	B	0.625–12.5	1/2.5	[31]
52	Spain	4.5	3.5	48.2	59.0	21.0	20.0	2.50	0.83	A	1.90	B	0.625–12.5	1/2.5	[31]
53	Spain	4.5	3.7	60.4	61.0	20.0	19.0	1.30	1.00	A	1.40	B	0.625–12.5	1/2.5	[31]
54	Spain	4.4	2.8	87.8	55.0	22.0	23.0	1.39	0.91	A	1.20	B	0.625–12.5	1/2.5	[31]
55	Spain	4.0	10.9	116.4	49.0	19.0	32.0	NA	NA	–	12.00	b	0.625–12.5	1/2.5	[31]
56	Brazil	5.0	0.9	19.3	91.1	1.8	6.2	0.45	1.06	A	0.46	B	4.0–75.0	1/1	[87]
57	Brazil	4.9	1.7	52.7	14.9	30.2	54.6	2.60	1.28	A	2.66	b	4.0–75.0	1/1	[87]
58	Brazil	4.1	1.4	51.9	52.9	10.5	36.2	1.30	1.12	a	1.32	B	4.0–75.0	1/1	[87]
59	Brazil	4.4	1.9	66.0	43.5	7.0	49.2	2.00	1.20	A	1.96	B	4.0–75.0	1/1	[87]
60	China	6.1	1.9	192.0	23.6	55.9	20.5	NA	NA	–	3.41	B	0.5–10	1/5	[60]
61	China	7.6	0.5	70.0	37.0	58.8	4.3	NA	NA	–	1.54	B	0.5–10	1/5	[60]
62	China	6.1	0.3	NA	75.6	21.6	2.8	NA	NA	–	2.20	b	0.1–1	1/1.5	[88]
63	China	7.4	0.4	NA	1.4	75.2	23.4	1.27	1.22	a	1.47	b	0.1–1	1/1.5	[88]
64	China	7.3	4.0	327.0	9.8	62.8	27.4	4.86	1.25	a	3.59	B	0.5–10	1/5	[89]
65	China	5.3	0.4	147.0	22.8	26.9	49.4	0.70	1.22	a	0.49	b	0.5–10	1/5	[89]
66	China	7.3	2.4	219.0	18.1	52.3	29.6	1.85	1.37	A	1.13	B	0.5–10	1/5	[89]
67	China	7.4	0.5	740.0	55.2	34.0	10.8	0.85	1.28	A	0.56	b	0.5–10	1/5	[89]
68	China	7.3	1.7	125.0	32.8	47.8	19.4	2.36	1.27	A	1.63	B	0.5–10	1/5	[89]
69	China	8.0	2.8	191.0	79.6	19.3	1.2	1.00	0.75	A	0.43	b	1.0–25	1/10	[90]
70	UK	6.0	4.1	251.1	22.0	51.0	27.0	NA	NA	–	2.36	B	0.01–0.04	NA	[74]
71	UK	5.3	3.2	142.1	33.0	38.0	29.0	NA	NA	–	1.44	B	0.01–0.04	NA	[74]
72	UK	6.7	3.5	242.8	25.0	55.0	20.0	NA	NA	–	1.78	B	0.01–0.04	NA	[74]

73	UK	4.8	1.3	44.2	17.0	65.0	18.0	NA	NA	–	1.65	B	0.01–0.04	NA	[74]
74	UK	5.2	1.5	49.4	77.0	13.0	10.0	NA	NA	–	2.23	B	0.01–0.04	NA	[74]
75	UK	7.4	1.9	197.5	35.0	39.0	26.0	NA	NA	–	1.14	b	0.01–0.04	NA	[74]
76	UK	7.1	1.8	196.1	15.0	40.0	45.0	NA	NA	–	1.87	B	0.01–0.04	NA	[74]
77	UK	7.4	1.4	115.2	66.0	20.0	14.0	NA	NA	–	3.13	B	0.01–0.04	NA	[74]
78	UK	7.4	1.2	103.2	71.0	17.0	12.0	NA	NA	–	2.95	B	0.01–0.04	NA	[74]
79	UK	7.2	4.5	217.4	27.0	52.0	21.0	NA	NA	–	4.21	B	0.01–0.04	NA	[74]
80	UK	6.7	3.3	117.7	63.0	20.0	17.0	1.54	0.88	A	NA	–	0.01–0.04	NA	[74]
81	UK	7.4	3.5	202.6	59.0	20.0	21.0	NA	NA	A	4.37	b	0.01–0.04	NA	[74]
82	China	7.3	1.0	106.0	43.5	21.0	35.5	3.03	0.88	a	NA	–	1–50	1/10	[35]
83	China	8.0	1.6	181.2	30.0	66.0	4.0	0.66	1.16	a	0.45	B	1.0–25	1/10	[91]
84	China	7.5	0.4	60.0	36.0	40.0	24.0	1.47	2.04	A	0.37	B	0.5–20	1/10	[83]
85	China	4.4	0.9	125.0	12.9	44.6	42.6	3.22	0.85	a	NA	–	10–50	1/20	[92]
86	China	6.8	0.2	180.3	50.7	37.5	11.8	1.45	0.71	A	0.60	B	10–50	1/20	[93]
87	China	4.0	1.0	96.9	16.9	67.2	15.9	1.42	1.10	A	1.46	B	0.05–1	1/25	[84]
88	China	5.8	6.1	312.0	11.3	73.1	15.6	1.76	1.47	a	1.96	b	0.05–1	1/25	[84]
89	China	6.2	5.0	180.0	4.8	75.8	19.4	2.02	1.05	a	2.05	B	0.05–1	1/25	[84]
90	China	6.5	1.1	179.9	31.4	60.0	8.6	0.58	2.17	a	0.91	B	0.1–25	1/2.5	[45]
91	China	4.3	1.0	87.1	30.4	21.8	47.8	1.27	1.02	A	2.20	B	0.1–25	1/2.5	[45]
92	China	6.8	1.4	58.4	7.8	89.7	2.6	NA	NA	A	2.17	B	1–4	1/25	[95]
93	China	7.8	0.7	NA	6.4	28.1	28.0	NA	NA	–	2.28	b	20–600	1/5	[96]
94	China	8.6	0.2	212.1	36.0	40.0	24.0	2.05	0.28	A	NA	–	1.0–25	1/10	[86]
95	India	4.8	0.8	11.0	23.5	34.0	42.5	1.50	0.91	A	1.58	B	0.005–5	1/2	[94]
96	India	5.2	1.2	10.0	72.0	12.5	15.5	2.40	0.83	A	2.32	B	0.005–5	1/2	[94]
97	India	4.4	0.5	7.4	68.2	12.4	19.4	NA	NA	–	0.09	B	0.005–5	1/2	[94]
98	Brazil	3.7	1.2	35.7	77.9	4.0	18.1	NA	NA	–	1.20	B	1	1/2	[14]

99	Brazil	6.9	9.6	52.2	10.9	20.7	68.4	NA	NA	–	5.70	B	1	1/2	[14]
100	Brazil	5.1	0.7	32.0	84.0	10.0	6.0	NA	NA	–	0.80	B	1	1/2	[14]
101	Brazil	3.8	1.0	27.6	88.0	4.0	8.0	NA	NA	–	1.10	B	1	1/2	[14]
102	Brazil	4.8	2.6	153.2	51.2	34.6	14.2	NA	NA	–	3.20	B	1	1/2	[14]
103	Brazil	5.1	5.4	125.2	47.3	18.2	34.5	NA	NA	–	4.60	B	1	1/2	[14]
104	Brazil	3.9	21.3	109.9	14.4	38.0	47.6	NA	NA	–	12.70	b	1	1/2	[14]
105	Brazil	3.7	2.9	56.9	65.6	10.1	24.3	NA	NA	–	1.30	B	1	1/2	[14]
106	Brazil	4.8	2.2	100.0	44.9	34.7	20.4	NA	NA	–	3.10	B	1	1/2	[14]
107	Brazil	4.5	1.0	39.2	72.0	24.0	4.0	NA	NA	–	0.90	B	1	1/2	[14]
108	Brazil	5.4	5.8	207.8	20.6	25.1	54.3	NA	NA	–	7.80	b	1	1/2	[14]

[1] Sub-datasets "A" and "a" were used for development and validation of the models for estimating K_f, respectively.

[2] Sub-datasets "B" and "b" were used for development and validation of the models for estimating K_d, respectively.

[3] $CaCl_2$ was used as background electrolyte in all batch sorption tests.

[4] "NA" means that the value was not reported in the cited reference.

[5] "–" denotes an exclusion from any sub-datasets.

Table S5. SMX sorption parameters and associated soil properties.

No.	Origin	Soil properties						Freundlich			Linear		Initial antibiotic concentration range [3]	Solid/Liquid ratio	Reference
		pH	OC	CEC	Sand	Silt	Clay	K_f	n	Sub-dataset [1]	K_d	Sub-dataset [2]			
			%	mmol kg⁻¹	%	%	%	$mg^{1-1/n}$ $L^{1/n}$ kg^{-1}			L kg⁻¹		mg L⁻¹	g mL⁻¹	
1	China	6.1	1.9	192.0	23.6	55.9	20.5	NA [4]	NA	– [5]	2.41	b	0.5–10	1/5	[60]
2	China	7.6	0.5	70.0	37.0	58.8	4.3	NA	NA	–	1.13	B	0.5–10	1/5	[60]
3	Czech	7.4	3.2	315.0	39.5	45.8	14.7	0.78	2.06	A	NA	–	0.5–10	1/2	[10]
4	Czech	6.3	1.7	218.0	17.7	76.4	5.9	1.44	2.28	A	0.51	B	0.5–10	1/2	[10]
5	Czech	7.2	2.1	261.0	29.3	64.2	6.5	0.91	1.79	a	0.40	B	0.5–10	1/2	[10]
6	Czech	5.9	1.2	92.6	76.5	21.4	2.1	1.60	2.13	A	0.61	B	0.5–10	1/2	[10]
7	Czech	5.3	1.2	166.0	20.2	75.4	4.4	0.62	1.79	A	0.26	B	0.5–10	1/2	[10]
8	Czech	5.7	0.9	155.0	15.9	76.7	7.4	1.25	1.60	a	0.63	B	0.5–10	1/2	[10]
9	Czech	4.4	1.6	175.0	44.2	48.3	7.5	3.19	1.27	A	2.41	B	0.5–10	1/2	[10]
10	Czech	5.1	2.6	155.0	54.0	43.9	2.1	2.85	1.34	A	1.99	B	0.5–10	1/2	[10]
11	Czech	4.4	1.7	132.0	38.2	56.5	5.3	3.74	1.14	a	3.25	B	0.5–10	1/2	[10]
12	Czech	4.7	2.2	186.0	39.1	58.4	2.6	4.21	1.38	A	3.02	B	0.5–10	1/2	[10]
13	Czech	4.3	0.7	45.5	84.0	11.0	5.0	1.14	1.40	A	0.68	b	0.5–10	1/2	[10]
14	Czech	7.4	0.4	191.0	44.2	48.3	7.5	0.13	1.71	A	NA	–	0.5–10	1/2	[10]
15	Czech	8.5	0.1	10.6	100.0	0.0	0.0	0.18	1.58	A	NA	–	0.5–10	1/2	[10]
16	UK	6.0	4.1	251.1	22.0	51.0	27.0	2.75	0.95	A	4.75	B	0.01–0.04	NA	[74]
17	UK	5.3	3.2	142.1	33.0	38.0	29.0	NA	NA	–	4.60	B	0.01–0.04	NA	[74]
18	UK	4.8	1.3	44.2	17.0	65.0	18.0	NA	NA	–	4.00	b	0.01–0.04	NA	[74]
19	UK	5.2	1.5	49.4	77.0	13.0	10.0	3.15	0.80	A	NA	–	0.01–0.04	NA	[74]

20	UK	7.4	1.9	197.5	35.0	39.0	26.0	NA	NA	–	0.87	B	0.01–0.04	NA	[74]
21	UK	7.1	1.8	196.1	15.0	40.0	45.0	1.10	1.03	A	0.67	B	0.01–0.04	NA	[74]
22	UK	7.4	1.4	115.2	66.0	20.0	14.0	NA	NA	–	1.07	B	0.01–0.04	NA	[74]
23	UK	7.4	1.2	103.2	71.0	17.0	12.0	NA	NA	–	0.78	B	0.01–0.04	NA	[74]
24	UK	7.2	4.5	217.4	27.0	52.0	21.0	8.95	0.78	a	2.03	B	0.01–0.04	NA	[74]
25	UK	6.7	3.3	117.7	63.0	20.0	17.0	2.73	1.00	A	2.34	B	0.01–0.04	NA	[74]
26	UK	7.4	3.5	202.6	59.0	20.0	21.0	1.07	0.92	A	2.09	B	0.01–0.04	NA	[74]
27	China	5.1	4.3	276.0	40.5	45.5	14.0	12.06	1.35	a	4.46	B	1–50	1/10	[79]
28	China	7.3	1.0	106.0	43.5	21.0	35.5	2.80	0.92	a	NA	–	1–50	1/10	[79]
29	China	5.1	4.3	276.0	40.5	45.5	14.0	12.60	1.35	a	4.46	B	1–50	1/10	[35]
30	China	7.3	1.0	106.0	43.5	21.0	35.5	2.80	0.92	A	NA	–	1–50	1/10	[35]
31	Czech	5.6	0.8	140.9	14.2	63.2	22.7	1.25	1.60	A	0.04	B	500–10000	1/2	[97]
32	Czech	7.2	1.1	174.7	16.1	63.7	20.2	0.62	1.79	A	0.02	B	500–10000	1/2	[97]
33	Czech	5.0	1.4	190.3	36.7	45.0	18.3	3.19	1.27	A	0.49	B	500–10000	1/2	[97]
34	Korea	5.7	2.2	45.1	74.5	20.0	5.5	1.31	1.05	A	1.27	B	5–10	1/5	[20]
35	Korea	5.5	4.0	60.5	65.2	26.7	8.1	1.18	1.11	A	1.10	B	5–10	1/5	[20]
36	Brazil	3.7	1.2	35.7	77.9	4.0	18.1	NA	NA	–	1.10	B	NA	1/2	[14]
37	Brazil	6.9	9.6	52.2	10.9	20.7	68.4	NA	NA	–	3.80	b	NA	1/2	[14]
38	Brazil	5.1	0.7	32.0	84.0	10.0	6.0	NA	NA	–	0.70	B	NA	1/2	[14]
39	Brazil	5.7	4.1	217.5	18.6	44.8	36.6	NA	NA	–	8.20	b	NA	1/2	[14]
40	Brazil	3.8	1.0	27.6	88.0	4.0	8.0	NA	NA	–	1.00	B	NA	1/2	[14]
41	Brazil	4.8	2.6	153.2	51.2	34.6	14.2	NA	NA	–	3.70	B	NA	1/2	[14]
42	Brazil	5.1	5.4	125.2	47.3	18.2	34.5	NA	NA	–	5.40	b	NA	1/2	[14]
43	Brazil	3.9	21.3	109.9	14.4	38.0	47.6	NA	NA	–	28.50	b	NA	1/2	[14]
44	Brazil	3.7	2.9	56.9	65.6	10.1	24.3	NA	NA	–	2.70	b	NA	1/2	[14]
45	Brazil	4.8	2.2	100.0	44.9	34.7	20.4	NA	NA	–	3.80	b	NA	1/2	[14]

46	Brazil	4.5	1.0	39.2	72.0	24.0	4.0	NA	NA	–	1.10	b	NA	1/2	[14]
47	Brazil	5.4	5.8	207.8	20.6	25.1	54.3	NA	NA	–	7.40	b	NA	1/2	[14]
48	Europe	8.1	2.9	273.0	27.2	52.2	20.7	0.65	1.44	A	0.36	B	0.5–10	1/2	[98]
49	Europe	8.1	1.8	235.0	5.4	58.1	36.5	0.37	1.20	A	0.27	B	0.5–10	1/2	[98]
50	Europe	7.5	1.4	165.0	16.5	66.5	17.0	0.39	1.26	A	0.26	B	0.5–10	1/2	[98]
51	Europe	7.3	1.1	118.0	14.7	72.9	12.4	0.84	1.23	A	0.60	B	0.5–10	1/2	[98]
52	Europe	5.8	1.9	183.0	40.4	41.3	18.3	3.21	1.26	A	2.44	B	0.5–10	1/2	[98]
53	Europe	5.8	2.2	196.0	22.9	57.7	19.4	4.24	1.20	A	3.53	B	0.5–10	1/2	[98]
54	Europe	5.4	0.6	38.0	85.4	7.0	7.6	0.99	1.22	A	0.72	B	0.5–10	1/2	[98]
55	New Zealand	4.3	2.1	154.0	11.0	62.0	27.0	2.24	0.80	A	1.38	b	1.5–15	NA	[99]
56	New Zealand	5.1	5.0	223.0	9.0	54.0	37.0	4.21	0.91	A	3.46	B	1.5–15	NA	[99]
57	New Zealand	5.4	8.2	356.0	34.0	48.0	17.0	6.75	0.60	A	3.86	B	1.5–15	NA	[99]
58	New Zealand	6.1	2.1	154.0	11.0	62.0	27.0	0.34	0.83	A	0.56	B	1.5–15	1/15	[58]
59	New Zealand	5.8	4.0	215.0	19.0	51.0	30.0	2.00	1.14	A	1.52	B	1.5–15	1/15	[58]
60	New Zealand	6.1	2.1	154.0	11.0	27.0	62.0	2.37	1.33	A	1.40	B	1.5–15	1/15	[12]
61	New Zealand	6.7	5.0	223.0	9.0	37.0	54.0	3.26	1.04	A	2.91	B	1.5–15	1/15	[12]
62	New Zealand	5.8	4.0	215.0	19.0	30.0	51.0	5.44	1.79	A	2.20	B	1.5–15	1/15	[12]
63	New Zealand	5.7	8.2	356.0	34.0	17.0	48.0	6.75	1.67	a	3.09	b	1.5–15	1/15	[12]
64	New Zealand	5.1	3.3	97.0	87.0	2.0	11.0	3.85	1.96	A	1.27	B	1.5–15	1/15	[12]
65	New Zealand	6.9	1.1	76.0	45.0	14.0	41.0	4.16	2.38	A	1.12	B	1.5–15	1/15	[12]
66	New Zealand	6.1	2.1	NA	11.0	62.0	27.0	NA	NA	–	1.40	b	1.5–15	1/15	[100]
67	China	4.0	1.0	96.9	16.9	67.2	15.9	1.36	1.05	A	1.38	B	0.05–1	1/25	[84]
68	China	5.8	6.1	312.0	11.3	73.1	15.6	2.60	1.08	A	2.66	B	0.05–1	1/25	[84]
69	China	6.2	5.0	180.0	4.8	75.8	19.4	2.26	1.04	a	2.29	B	0.05–1	1/25	[84]
70	China	7.6	2.4	106.0	16.5	74.3	9.2	0.58	0.96	A	0.57	B	0.05–1	1/25	[84]
71	China	5.6	1.0	218.0	2.4	82.9	14.7	0.65	1.20	A	0.68	B	0.05–1	1/25	[84]

72	China	6.9	1.6	222.7	43.7	27.4	28.9	0.72	1.33	A	0.45	B	1–10	1/2.5	[101]
73	China	4.5	1.1	224.2	24.5	39.7	35.8	1.86	1.51	A	1.04	B	1–10	1/2.5	[101]
74	China	5.7	11.3	538.1	46.7	28.2	25.1	10.35	1.46	A	8.47	b	1–10	1/2.5	[101]
75	China	5.0	1.2	116.2	32.6	33.2	34.2	3.42	0.84	A	2.46	B	0.5–10	1/10	[102]
76	Australia	5.3	4.3	4.8	67.0	10.0	23.0	4.08	0.91	A	4.53	B	0.5–10	1/2.5	[103]
77	Australia	6.0	1.2	5.9	72.0	12.0	15.0	1.03	0.99	a	1.05	B	0.5–10	1/2.5	[103]
78	Australia	4.7	1.6	3.4	75.0	10.0	14.0	1.66	1.00	A	1.66	B	0.5–10	1/2.5	[103]
79	China	8.6	0.2	212.1	36.0	40.0	24.0	0.71	0.48	A	NA	–	1.0–25	1/10	[86]

[1] Sub-datasets "A" and "a" were used for development and validation of the models for estimating K_f, respectively.

[2] Sub-datasets "B" and "b" were used for development and validation of the models for estimating K_d, respectively.

[3] $CaCl_2$ was used as background electrolyte in all batch sorption tests.

[4] "NA" means that the value was not reported in the cited reference.

[5] "–" denotes an exclusion from any sub-datasets.

Table S6. OTC sorption parameters and associated soil properties.

No.	Origin	Soil properties						Freundlich			Linear		Initial antibiotic concentration range [3]	Solid/Liquid ratio	Reference
		pH	OC	CEC	Sand	Silt	Clay	K_f	n	Sub-dataset [1]	K_d	Sub-dataset [2]			
			%	mmol kg^{-1}		%		mg$^{1-1/n}$ L$^{1/n}$ kg^{-1}			L kg^{-1}		mg L^{-1}	g mL^{-1}	
1	China	5.5	6.1	13.7	29.2	45.2	25.6	907.82	1.49	a	1009.25	b	1.0–25.0	1/50	[104]
2	China	5.5	6.1	13.7	29.2	45.2	25.6	907.82	1.49	a	1009.25	b	1.0–25.0	1/50	[105]
3	China	5.5	6.1	13.7	29.2	45.2	25.6	1489.36	1.22	a	1749.85	B	1.0–25.0 [4]	1/50	[105]
4	China	5.5	6.1	13.7	29.2	45.2	25.6	807.24	1.21	a	841.40	b	1.0–25.0 [5]	1/50	[105]
5	China	7.1	1.2	154.2	13.4	39.5	47.1	855.07	1.18	A	547.02	B	1–10	1/50	[106]
6	China	6.5	1.4	163.2	17.6	37.1	45.3	529.66	1.17	A	516.42	B	1–10	1/50	[106]
7	China	6.2	1.0	285.6	NA [6]	NA	NA	1391.55	1.27	a	NA	–	2–80	1/100	[107]
8	China	NA	1.2	307.8	NA	NA	NA	1508.04	1.46	a	784.36	b	1–40	1/250	[108]
9	Spain	5.0	3.1	129.3	58.0	22.0	20.0	3495.00	2.70	A	1043.80	B	2.395–191.6	1/40	[13]
10	Spain	5.1	3.0	98.5	64.0	17.0	19.0	1882.00	2.08	A	485.71	B	2.395–191.6	1/40	[13]
11	Spain	4.3	3.4	59.2	58.0	19.0	22.0	2783.00	2.56	A	671.34	B	2.395–191.6	1/40	[13]
12	Spain	4.5	2.8	53.1	61.0	18.0	20.0	4043.00	3.03	A	1243.90	B	2.395–191.6	1/40	[13]
13	Spain	4.4	2.4	58.9	62.0	20.0	17.0	3765.00	2.70	A	1260.70	B	2.395–191.6	1/40	[13]
14	Spain	4.0	1.7	52.1	53.0	27.0	20.0	3749.00	6.25	A	219.29	B	2.395–191.6	1/40	[13]
15	Spain	3.8	2.0	47.4	60.0	19.0	21.0	3563.00	3.23	A	766.99	B	2.395–191.6	1/40	[13]
16	Spain	4.7	3.9	65.9	51.0	21.0	29.0	3932.00	1.89	A	NA	–	2.395–191.6	1/40	[13]
17	Spain	4.9	3.8	64.8	65.0	17.0	19.0	3312.00	2.13	A	1426.20	B	2.395–191.6	1/40	[13]
18	Spain	4.4	3.7	51.3	62.0	20.0	19.0	2528.00	2.70	A	473.27	b	2.395–191.6	1/40	[13]
19	Spain	4.2	2.4	42.5	65.0	15.0	21.0	2198.00	2.86	A	297.12	B	2.395–191.6	1/40	[13]

20	Spain	4.1	1.2	44.7	64.0	19.0	17.0	2811.00	3.13	A	431.24	B	2.395–191.6	1/40	[13]
21	Spain	4.2	2.5	46.5	66.0	16.0	18.0	3625.00	3.03	A	916.47	B	2.395–191.6	1/40	[13]
22	Spain	3.9	1.7	52.1	43.0	27.0	30.0	2827.00	3.03	A	471.42	B	2.395–191.6	1/40	[13]
23	Spain	4.4	1.3	38.8	66.0	15.0	18.0	2787.00	2.94	A	488.50	B	2.395–191.6	1/40	[13]
24	Spain	4.1	1.3	40.0	68.0	14.0	17.0	2098.00	2.94	A	247.95	B	2.395–191.6	1/40	[13]
25	Spain	3.8	2.7	63.6	54.0	24.0	21.0	2456.00	2.86	A	384.89	B	2.395–191.6	1/40	[13]
26	Spain	3.7	1.7	44.4	59.0	22.0	20.0	2552.00	3.03	A	364.32	B	2.395–191.6	1/40	[13]
27	Spain	4.3	1.1	40.8	70.0	12.0	18.0	1648.00	2.94	A	148.71	B	2.395–191.6	1/40	[13]
28	Spain	4.0	1.6	47.4	55.0	22.0	23.0	2394.00	2.78	A	389.08	B	2.395–191.6	1/40	[13]
29	Spain	4.0	1.9	46.1	58.0	23.0	19.0	2753.00	3.03	A	440.53	B	2.395–191.6	1/40	[13]
30	Spain	4.4	2.1	52.9	61.0	16.0	23.0	3223.00	2.44	A	1048.60	B	2.395–191.6	1/40	[13]
31	Spain	4.1	2.1	52.5	55.0	26.0	19.0	3530.00	3.03	A	851.75	B	2.395–191.6	1/40	[13]
32	Spain	3.8	1.6	41.5	57.0	25.0	18.0	2920.00	3.85	A	289.16	B	2.395–191.6	1/40	[13]
33	Spain	4.0	2.0	43.0	64.0	19.0	17.0	1971.00	2.94	A	215.96	B	2.395–191.6	1/40	[13]
34	Spain	4.2	1.1	42.4	69.0	13.0	18.0	3312.00	4.35	A	314.09	B	2.395–191.6	1/40	[13]
35	Spain	4.5	1.1	42.5	67.0	14.0	19.0	2790.00	3.45	A	332.48	B	2.395–191.6	1/40	[13]
36	Spain	4.5	5.3	116.5	41.0	26.0	34.0	4747.00	1.72	A	NA	–	2.395–191.6	1/40	[13]
37	Spain	4.2	3.3	65.1	57.0	20.0	23.0	3772.00	3.03	A	1023.50	B	2.395–191.6	1/40	[13]
38	Spain	6.2	1.7	164.7	56.0	20.0	24.0	1218.00	2.50	A	130.24	B	2.395–191.6	1/40	[13]
39	Spain	3.7	5.0	76.9	51.0	22.0	27.0	3092.00	2.13	A	1238.70	B	2.395–191.6	1/40	[13]
40	Spain	4.1	1.8	44.9	48.0	23.0	29.0	3769.00	3.57	A	731.49	B	2.395–191.6	1/40	[13]
41	Spain	4.5	1.3	43.2	63.0	17.0	19.0	2775.00	3.57	A	300.86	B	2.395–191.6	1/40	[13]
42	Spain	4.3	1.8	49.5	62.0	22.0	16.0	4435.00	2.78	A	NA	–	2.395–191.6	1/40	[13]
43	Spain	4.3	2.5	63.9	59.0	19.0	22.0	4376.00	3.03	A	NA	–	2.395–191.6	1/40	[13]
44	Spain	4.2	2.2	54.5	63.0	20.0	17.0	4092.00	3.70	A	883.13	B	2.395–191.6	1/40	[13]
45	Spain	4.2	2.3	43.9	65.0	18.0	17.0	2024.00	2.33	A	423.90	B	2.395–191.6	1/40	[13]

46	Spain	4.4	2.5	54.0	62.0	20.0	18.0	2349.00	2.33	A	578.40	B	2.395–191.6	1/40	[13]
47	Spain	4.1	3.1	53.3	65.0	14.0	21.0	3860.00	2.86	A	1217.40	B	2.395–191.6	1/40	[13]
48	Spain	4.5	3.7	67.3	63.0	18.0	19.0	4168.00	2.70	A	1636.90	B	2.395–191.6	1/40	[13]
49	Spain	4.1	2.7	50.3	48.0	31.0	21.0	4375.00	3.13	A	1481.70	B	2.395–191.6	1/40	[13]
50	Spain	4.2	3.3	54.5	49.0	29.0	22.0	4671.00	2.70	A	NA	–	2.395–191.6	1/40	[13]
51	Spain	4.5	3.5	48.2	59.0	21.0	20.0	2945.00	2.22	A	1031.30	B	2.395–191.6	1/40	[13]
52	Spain	4.5	3.7	60.4	61.0	20.0	19.0	5110.00	3.45	A	NA	–	2.395–191.6	1/40	[13]
53	Spain	4.4	2.8	87.8	55.0	22.0	23.0	3387.00	2.38	A	1229.00	B	2.395–191.6	1/40	[13]
54	Spain	6.4	4.6	303.1	44.0	39.0	18.0	1833.00	1.85	a	617.39	b	2.395–191.6	1/40	[13]
55	Spain	5.9	2.0	149.6	22.0	62.0	16.0	1015.00	1.85	A	227.56	B	2.395–191.6	1/40	[13]
56	Spain	4.5	3.0	67.6	39.0	47.0	14.0	2878.00	2.04	A	1158.30	B	2.395–191.6	1/40	[13]
57	Spain	5.5	2.8	110.9	35.0	45.0	20.0	2255.00	1.92	A	821.60	B	2.395–191.6	1/40	[13]
58	Spain	6.2	2.9	234.1	26.0	51.0	23.0	1695.00	1.67	A	691.55	B	2.395–191.6	1/40	[13]
59	Spain	5.1	4.6	167.7	37.0	45.0	19.0	3976.00	1.61	A	NA	–	2.395–191.6	1/40	[13]
60	Spain	5.2	1.5	67.5	30.0	55.0	15.0	3326.00	2.50	A	1075.60	B	2.395–191.6	1/40	[13]
61	Spain	5.7	2.3	128.6	26.0	58.0	16.0	1410.00	2.00	A	318.39	B	2.395–191.6	1/40	[13]
62	Spain	4.3	4.3	60.4	41.0	42.0	17.0	3008.00	2.27	A	1032.40	B	2.395–191.6	1/40	[13]
63	Spain	6.4	1.7	125.4	27.0	56.0	17.0	1760.00	2.08	A	427.84	B	2.395–191.6	1/40	[13]
64	Spain	5.4	2.0	144.5	25.0	60.0	15.0	2139.00	2.00	A	682.82	B	2.395–191.6	1/40	[13]
65	Spain	5.0	3.6	86.2	25.0	59.0	17.0	2186.00	2.86	A	NA	–	2.395–191.6	1/40	[13]
66	Spain	5.5	1.6	80.3	30.0	47.0	23.0	2157.00	2.56	A	377.24	B	2.395–191.6	1/40	[13]
67	Spain	4.3	3.4	59.0	54.7	26.0	19.3	906.49	2.08	A	NA	–	23.02–276.24	1/40	[109]
68	Spain	4.3	1.1	41.0	64.7	14.0	21.3	551.63	2.94	A	16.76	B	23.02–276.24	1/40	[109]
69	Spain	4.0	10.9	116.0	58.7	16.0	25.3	4420.12	5.88	a	NA	–	23.02–276.24	1/40	[109]
70	Spain	5.9	2.0	150.0	29.3	49.3	21.4	593.83	1.79	A	NA	–	23.02–276.24	1/40	[109]
71	Spain	6.4	1.8	125.0	27.3	51.3	21.4	778.76	1.85	A	70.85	B	23.02–276.24	1/40	[109]

72	Spain	5.4	6.9	174.0	61.3	23.3	15.4	2560.60	2.22	a	NA	–	23.02–276.24	1/40	[109]
73	China	7.1	0.4	60.3	24.0	40.0	36.0	351.43	1.03	A	375.59	B	1–25	1/10	[110]
74	China		0.2	204.3	36.0	40.0	24.0	NA	NA	–	356.99	b	1–25	1/10	[48]
75	USA	7.1	2.2	233.0	NA	NA	22.3	NA	NA	–[7]	847.15	b	2.30215–30.215	1/100	[44]
76	USA	7.0	3.0	268.0	NA	NA	24.5	2138.00	1.43	a	847.15	b	2.30215–30.215	1/100	[44]
77	USA	6.7	2.3	254.0	NA	NA	25.3	NA	NA	–	1252.50	b	2.30215–30.215	1/100	[44]
78	USA	6.6	1.3	138.0	NA	NA	19.9	1819.75	1.64	a	452.96	b	2.30215–30.215	1/100	[44]
79	USA	5.2	1.3	90.0	NA	NA	15.8	1949.90	1.79	a	379.55	b	2.30215–30.215	1/100	[44]
80	USA	4.7	2.3	151.0	NA	NA	19.0	3162.37	1.75	a	851.26	b	2.30215–30.215	1/100	[44]
81	USA	6.3	1.9	180.0	NA	NA	21.1	2238.77	1.52	a	782.14	b	2.30215–30.215	1/100	[44]
82	USA	5.8	2.2	235.0	NA	NA	24.7	2630.31	NA	a	782.14	B	2.30215–30.215	1/100	[44]
83	USA	5.6	3.1	104.2	16.9	70.7	12.4	NA	NA	–	1454.00	B	NA	NA	[2]
84	USA	5.4	0.2	60.8	62.7	28.5	8.9	NA	NA	–	781.00	B	NA	NA	[2]
85	USA	4.1	1.8	18.2	94.0	4.2	1.8	NA	NA	–	486.00	B	NA	NA	[2]
86	USA	3.8	1.7	8.2	94.9	3.8	1.4	NA	NA	–	618.00	B	NA	NA	[2]
87	USA	3.6	0.8	33.0	57.6	41.2	1.2	NA	NA	–	771.00	B	NA	NA	[2]
88	USA	5.5	4.1	78.8	71.9	24.6	3.5	NA	NA	–	1837.00	b	NA	NA	[2]
89	USA	7.2	3.9	439.5	14.4	35.9	49.7	NA	NA	–	1751.00	B	NA	NA	[2]
90	Korea	5.6	1.1	103.5	25.9	58.5	15.6	3012.40	1.14	A	NA	–	1–40	1/10	[71]
91	Korea	8.1	0.5	52.0	56.2	38.9	4.9	1965.20	0.98	A	NA	–	1–40	1/10	[71]
92	China	4.0	15.5	187.7	NA	NA	48.0	NA	NA	A	2191.00	b	3.6832–118.7832	1/200	[51]
93	China	4.5	10.0	182.2	NA	NA	46.3	NA	NA	A	888.00	b	3.6832–118.7832	1/200	[51]
94	China	7.1	4.4	NA	NA	NA	48.2	NA	NA	A	769.00	b	3.6832–118.7832	1/200	[51]
95	UK	6.8	3.1	224.0	42.6	32.3	25.1	1814.00	1.54	A	NA	–	NA	NA	[54]
96	UK	6.6	2.2	114.0	69.2	20.5	10.3	655.00	1.47	A	NA	–	NA	NA	[54]
97	UK	6.0	4.1	251.1	22.0	51.0	27.0	NA	NA	A	1433.00	B	0.01–0.04	NA	[29]

98	UK	5.3	3.2	142.1	33.0	38.0	29.0	NA	NA	A	1486.00	B	0.01–0.04	NA	[29]
99	UK	6.7	3.5	242.8	25.0	55.0	20.0	NA	NA	A	1087.00	B	0.01–0.04	NA	[29]
100	UK	7.1	1.8	196.1	15.0	40.0	45.0	203.30	0.37	A	NA	–	0.01–0.04	NA	[29]
101	UK	7.4	1.2	103.2	71.0	17.0	12.0	1013.00	0.33	A	NA	–	0.01–0.04	NA	[29]
102	China	6.9	0.7	352.0	NA	NA	NA	495.00	NA	a	NA	–	25–200	1/62.5	[23]
103	China	4.6	1.6	600.0	NA	NA	NA	1575.00	NA	a	NA	–	25–200	1/62.5	[23]
104	China	7. 9	1.9	104.0	76.6	6.3	17.1	1660.00	0.54	A	NA	–	2–10	1/100	[111]
105	China	8. 6	1.4	46.6	73.7	9.5	16.8	NA	NA	–	NA	–	2–10	1/100	[111]
106	China	8. 5	0.2	82.1	76.8	8.1	15.2	NA	NA	–	NA	–	2–10	1/100	[111]
107	China	8.2	1.7	138.7	4.6	32.4	63.0	NA	NA	–	767.01	b	NA	NA	[112]
108	China	6.8	1.6	178.0	7.3	59.2	33.5	395.37	1.11	A	385.48	B	5–20	1/25	[112]
109	China	7.1	2.7	282.0	6.1	65.3	28.6	545.76	1.07	a	544.50	B	5–20	1/25	[112]
110	China	9.0	0.3	210.5	25.8	27.4	46.8	1083.43	0.94	A	NA	–	5–30	1/25	[113]
111	China	6.1	1.0	107.5	33.3	52.2	14.5	1984.27	1.07	A	NA	–	5–30	1/25	[113]
112	China	6.8	0.6	71.2	73.2	18.6	8.2	665.12	0.81	A	578.36	B	5–30	1/25	[113]
113	China	7.4	0.3	209.0	NA	NA	34.1	290.00	1.39	a	119.00	b	2.0–80.0	1/50	[114]
114	China	5.3	0.7	124.0	46.2	33.6	20.2	1297.00	1.71	A	980.00	B	1.0–20	1/200	[115]
115	China	8.0	1.0	NA	NA	NA	NA	364.50	1.17	a	243.72	b	3.75–120	1/50	[116]
116	China	7.2	0.3	72.0	79.7	6.3	14.1	326.00	1.20	A	199.00	B	2.0–75	NA	[79]
117	China	8.5	0.2	82.1	76.8	8.1	15.2	335.00	1.15	A	228.00	B	2.0–75	NA	[79]
118	China	7.1	0.2	133.0	76.6	7.3	16.1	329.00	1.02	A	299.00	B	2.0–75	NA	[79]
119	Denmark	6.1	1.6	100.0	78.0	10.7	11.3	NA	NA	–	680.00	B	0.25–5.0	1/10	[117]
120	Denmark	5.6	1.4	67.0	90.0	4.8	5.2	NA	NA	–	670.00	B	0.25–5.0	1/10	[117]
121	Denmark	5.6	1.1	131.0	64.0	19.1	16.9	NA	NA	–	1026.00	B	0.25–5.0	1/10	[117]
122	Denmark	6.3	1.5	353.0	85.5	8.7	5.8	NA	NA	–	417.00	B	0.25–5.0	1/10	[117]
123	China	6.8	1.2	307.8	NA	NA	NA	1508.04	1.46	a	NA	–	20	1/250	[118]

124	China	7.6	0.6	64.0	38.6	39.7	21.7	74.00	1.15	A	52.34	B	1–40	1/40	[119]
125	China	4.3	0.8	257.0	46.8	42.9	10.3	2631.04	2.20	A	NA	–	1–40	1/40	[119]
126	China	3.5	2.5	74.0	49.2	42.3	8.5	1883.05	2.31	A	NA	–	1–40	1/40	[119]
127	China	NA	2.3	163.3	NA	NA	NA	NA	NA	A	404.94	b	10–100	1/62.5	[120]
129	China	7.6	0.9	133.0	16.6	73.8	9.6	187.00	1.17	A	151.90	B	1. 0–20.0 [5]	1/100	[121]
128	India	NA	2.9	265.0	NA	NA	21.0	1229.04	1.69	a	1229.00	b	NA	1/50	[11]
130	Spain	8.2	1.1	171.0	13.6	55.5	30.9	280.00	1.05	A	276.85	B	0.5–10	1/50	[6]
131	Spain	8.1	2.3	154.0	29.1	41.8	29.1	170.00	1.10	A	160.00	B	0.5–10	1/50	[6]
132	Spain	8.0	1.0	121.0	7.7	58.3	34.0	105.00	1.10	A	95.64	B	0.5–10	1/50	[6]
133	Spain	7.9	2.6	138.0	51.5	28.9	19.6	323.00	1.20	A	315.60	B	0.5–10	1/50	[6]
134	Spain	6.5	8.8	243.0	36.7	52.0	11.3	NA	NA	–	786.08	b	0.5–10	1/50	[6]
135	Spain	5.3	1.9	65.0	56.4	32.7	10.9	209.00	1.72	A	151.33	B	0.5–10	1/50	[6]
136	Spain	5.0	1.0	123.0	36.7	42.8	20.5	586.00	1.39	A	679.51	B	0.5–10	1/50	[6]
137	Spain	3.9	7.3	215.0	81.0	10.3	8.7	1362.00	1.30	a	1941.10	B	0.5–10	1/50	[6]
138	Spain	8.2	1.5	106.0	38.6	37.7	23.7	158.00	1.25	A	134.08	B	0.5–10	1/50	[6]
139	Spain	7.2	2.5	110.0	76.0	13.5	10.5	237.00	1.72	A	181.79	B	0.5–10	1/50	[6]
140	Spain	6.0	1.7	121.0	47.6	30.2	22.2	308.00	1.43	A	268.30	B	0.5–10	1/50	[6]
141	China	8.3	0.6	166.8	36.5	55.6	7.9	295.12	5.88	A	956.82	B	2.5–10.0	1/25	[122]
142	China	8.4	0.4	138.7	60.2	34.5	5.3	295.12	3.57	A	649.82	B	2.5–10.0	1/25	[122]
143	China	9.0	0.3	210.5	25.8	27.4	46.8	1083.43	0.94	A	NA	–	NA	NA	[123]
144	China	6.1	1.0	107.5	33.3	52.2	14.5	1984.27	1.07	A	NA	–	NA	NA	[123]
145	China	6.8	0.6	71.2	73.2	18.6	8.2	665.12	0.81	A	578.36	B	NA	NA	[123]
146	China	6.2	0.2	199.0	14.9	36.2	48.9	809.00	1.01	A	815.36	B	1.0–10	1/50	[124]
147	China	5.7	1.3	185.6	28.1	38.3	33.6	1218.99	1.25	A	532.11	B	5.0–200.0	1/50	[125]
148	China	6.3	1.1	151.2	20.2	42.3	37.5	1333.52	1.29	A	601.17	B	5.0–200.0	1/50	[125]
149	China	8.4	0.7	124.6	5.3	34.5	60.2	402.53	1.61	A	348.82	b	2–20	1/25	[126]

150	China	8.3	0.6	166.8	7.9	55.6	36.5	497.05	1.20	A	408.98	B	2–20	1/25	[126]
151	China	6.5	0.6	201.7	14.3	79.2	6.5	1551.67	1.73	A	NA	–	2–20	1/25	[126]
152	China	8.2	1.7	138.7	4.6	32.4	63.0	758.58	1.70	A	NA	–	2–20	1/25	[126]
153	China	6.7	0.4	190.3	1.2	21.7	77.2	478.08	2.05	a	NA	–	2–20	1/25	[126]
154	China	7.8	0.7	144.0	31.6	64.7	3.7	NA	NA	–	503.10	B	NA	NA	[127]
155	China	7.8	2.5	155.8	NA	NA	34. 0	613.76	0.36	a	NA	–	0. 5–10	1/5	[128]
156	China	5.6	2.0	134.5	NA	NA	34. 4	885.12	0.34	a	NA	–	0. 5–10	1/5	[128]
157	China	4.8	1.9	68.2	NA	NA	37. 2	928.97	0.34	a	NA	–	0. 5–10	1/5	[128]
158	China	NA	3.2	NA	NA	NA	NA	1316.52	1.18	a	1399.50	b	1–15	1/80	[129]
159	China	NA	0.6	NA	NA	NA	NA	278.01	1.22	a	224.90	b	1–15	1/80	[129]

[1] Sub-datasets "A" and "a" were used for development and validation of the models for estimating K_f, respectively.

[2] Sub-datasets "B" and "b" were used for development and validation of the models for estimating K_d, respectively.

[3] $CaCl_2$ was used as background electrolyte in all batch sorption tests, unless otherwise stated.

[4] KCl was used as background electrolyte in batch sorption test.

[5] NaCl was used as background electrolyte in batch sorption test.

[6] "NA" means that the value was not reported in the cited reference.

[7] "–" denotes an exclusion from any sub-datasets.

Table S7. TC sorption parameters and associated soil properties.

No.	Origin	Soil properties						Freundlich			Linear		Initial antibiotic concentration range [3]	Solid/Liquid ratio	Reference
		pH	OC	CEC	Sand	Silt	Clay	K_f	*n*	Sub-dataset [1]	K_d	Sub-dataset [2]			
			%	mmol kg⁻¹	%	%	%	$mg^{1-1/n}$ $L^{1/n}$ kg^{-1}			L kg^{-1}		mg L^{-1}	g mL^{-1}	
1	China	6.1	1.0	107.5	58.0	27.0	15.0	3163.92	1.61	A	NA [4]	– [5]	4.444–177.76	1/40	[130]
2	China	5.5	0.6	136.9	14.9	36.2	48.9	279.46	0.75	A	157.99	b	1.25–25.0	1/10	[131]
3	Spain	6.0	1.7	121.0	29.2	45.2	25.6	NA	NA	–	471.80	B	1.25–25.0	1/10	[131]
4	Spain	5.0	2.4	61.9	13.4	39.5	47.1	1786.49	0.93	A	1678.80	b	1–10	1/50	[106]
5	Spain	4.2	1.1	42.4	17.6	37.1	45.3	1577.61	0.89	A	1409.29	B	1–10	1/50	[106]
6	Spain	4.0	1.9	46.1	14.9	36.2	48.9	1465.55	1.56	A	1447.20	B	1–25	1/50	[40]
7	China	8.6	1.1	234.0	29.2	45.2	25.6	2648.50	1.10	A	NA	–	1–25	1/50	[40]
8	Spain	4.1	2.1	52.5	14.9	36.2	48.9	1479.11	0.91	A	NA	–	1–25	NA	[132]
9	China	7.2	1.1	135.7	29.2	45.2	25.6	2630.27	1.22	A	NA	–	1–25	NA	[132]
10	Spain	8.1	2.3	154.0	69.4	25.6	5.1	3330.00	3.33	a	NA	–	10–200	1/125	[17]
11	Spain	4.7	3.9	65.9	53.9	36.8	9.4	1120.00	2.70	A	NA	–	10–200	1/125	[17]
12	China	8.4	1.9	240.6	68.8	24.8	6.4	2700.00	2.17	A	NA	–	10–200	1/125	[17]
13	Spain	4.2	3.3	65.1	74.8	19.6	5.6	1640.00	1.96	A	NA	–	10–200	1/125	[17]
14	Spain	4.9	3.8	64.8	54.7	26.0	19.3	1125.69	2.63	A	70.37	B	23.02–276.24	1/40	[109]
15	Spain	5.4	6.9	174.0	64.7	14.0	21.3	756.18	3.85	A	17.99	B	23.02–276.24	1/40	[109]
16	Spain	5.3	1.9	65.0	58.7	16.0	25.3	4088.29	6.85	a	108.85	b	23.02–276.24	1/40	[109]
17	Spain	4.3	1.1	41.0	29.3	49.3	21.4	494.00	2.04	A	NA	–	23.02–276.24	1/40	[109]
18	China	6.2	2.2	198.8	27.3	51.3	21.4	890.95	2.27	A	74.85	b	23.02–276.24	1/40	[109]
19	Spain	5.4	2.0	144.5	61.3	23.3	15.4	2327.80	1.96	A	616.75	b	23.02–276.24	1/40	[109]

20	Spain	4.3	1.1	40.8	58.0	22.0	20.0	1405.16	2.22	A	235.84	B	2.222–177.76	1/40	[30]
21	China	5.5	0.6	136.9	64.0	17.0	19.0	931.04	1.85	A	206.91	B	2.222–177.76	1/40	[30]
22	Spain	3.8	1.6	41.5	59.0	19.0	22.0	1533.19	2.22	A	296.22	b	2.222–177.76	1/40	[30]
23	Spain	4.1	2.7	50.3	62.0	18.0	20.0	1900.05	2.70	a	277.35	B	2.222–177.76	1/40	[30]
24	Spain	4.1	3.1	53.3	63.0	20.0	17.0	1853.25	2.70	A	246.07	B	2.222–177.76	1/40	[30]
25	Spain	6.2	1.7	164.7	53.0	27.0	20.0	1640.61	2.04	A	403.64	B	2.222–177.76	1/40	[30]
26	Spain	4.5	3.5	48.2	60.0	19.0	21.0	1921.29	3.03	A	197.87	B	2.222–177.76	1/40	[30]
27	Spain	6.7	5.4	220.0	50.0	21.0	29.0	3785.78	2.04	a	NA	–	2.222–177.76	1/40	[30]
28	Spain	6.5	8.8	243.0	64.0	17.0	19.0	3948.04	2.13	a	NA	–	2.222–177.76	1/40	[30]
29	Spain	6.2	2.9	234.1	61.0	20.0	19.0	2201.26	3.23	A	260.02	B	2.222–177.76	1/40	[30]
30	Spain	4.3	2.5	63.9	64.0	15.0	21.0	1802.46	2.56	A	265.03	B	2.222–177.76	1/40	[30]
31	Spain	4.4	1.3	38.8	64.0	19.0	17.0	1248.40	2.27	A	201.11	B	2.222–177.76	1/40	[30]
32	Spain	4.7	2.6	71.8	66.0	16.0	18.0	1764.21	3.70	a	113.32	B	2.222–177.76	1/40	[30]
33	Spain	6.4	1.8	125.4	43.0	27.0	30.0	2326.34	3.13	A	300.00	B	2.222–177.76	1/40	[30]
34	Spain	4.1	1.8	44.9	67.0	15.0	18.0	1730.21	3.03	A	163.92	b	2.222–177.76	1/40	[30]
35	Spain	4.1	1.3	40.0	69.0	14.0	17.0	1330.73	3.57	A	69.47	b	2.222–177.76	1/40	[30]
36	Spain	3.7	1.7	44.4	55.0	24.0	21.0	1388.38	2.86	A	126.61	B	2.222–177.76	1/40	[30]
37	Spain	4.5	2.8	53.1	58.0	22.0	20.0	1085.08	2.63	a	92.63	B	2.222–177.76	1/40	[30]
38	Spain	4.6	3.8	68.2	70.0	12.0	18.0	1844.72	5.56	A	53.11	B	2.222–177.76	1/40	[30]
39	Spain	5.1	4.6	167.7	55.0	22.0	23.0	2250.29	3.13	A	265.89	B	2.222–177.76	1/40	[30]
40	Spain	4.2	3.3	54.5	58.0	23.0	19.0	1908.07	2.38	a	392.24	b	2.222–177.76	1/40	[30]
41	Spain	4.4	2.8	87.8	61.0	16.0	23.0	2039.54	2.44	A	408.97	b	2.222–177.76	1/40	[30]
42	Spain	4.3	4.3	60.4	55.0	26.0	19.0	2282.97	3.23	A	284.49	B	2.222–177.76	1/40	[30]
43	Spain	4.5	3.7	67.3	57.0	25.0	18.0	1895.70	3.57	A	154.44	B	2.222–177.76	1/40	[30]
44	Spain	4.3	3.4	59.2	64.0	19.0	17.0	1063.37	2.38	a	123.09	B	2.222–177.76	1/40	[30]
45	Spain	4.0	1.6	47.4	69.0	13.0	18.0	1424.68	2.78	A	145.84	B	2.222–177.76	1/40	[30]

46	Spain	4.0	2.0	43.0	67.0	14.0	19.0	1564.49	3.33	A	100.80	B	2.222–177.76	1/40	[30]
47	Spain	8.2	1.1	171.0	40.0	26.0	34.0	3319.22	1.64	A	NA	–	2.222–177.76	1/40	[30]
48	Spain	5.2	1.5	67.5	57.0	20.0	23.0	2253.75	3.13	A	297.30	B	2.222–177.76	1/40	[30]
49	Spain	5.5	1.6	80.3	56.0	20.0	24.0	2384.28	2.94	A	405.62	B	2.222–177.76	1/40	[30]
50	Spain	6.7	6.6	277.0	51.0	22.0	27.0	2971.58	3.23	A	593.16	B	2.222–177.76	1/40	[30]
51	Spain	5.5	2.8	110.9	48.0	23.0	29.0	2194.85	3.03	a	296.21	B	2.222–177.76	1/40	[30]
52	Spain	5.4	6.9	173.8	62.0	19.0	19.0	2435.80	2.70	A	483.12	B	2.222–177.76	1/40	[30]
53	Spain	4.5	3.7	60.4	52.0	22.0	26.0	1994.30	2.44	A	416.53	b	2.222–177.76	1/40	[30]
54	Spain	4.2	2.2	54.5	65.0	17.0	18.0	1805.92	2.27	A	418.44	b	2.222–177.76	1/40	[30]
55	Spain	4.4	2.5	54.0	62.0	22.0	16.0	1831.65	2.70	A	302.11	B	2.222–177.76	1/40	[30]
56	Spain	5.9	2.0	149.6	59.0	19.0	22.0	2115.77	2.44	a	489.56	B	2.222–177.76	1/40	[30]
57	Spain	4.2	2.3	43.9	63.0	20.0	17.0	1808.25	2.70	A	322.58	B	2.222–177.76	1/40	[30]
58	Spain	3.9	1.7	52.1	65.0	18.0	17.0	1221.31	2.33	A	202.46	B	2.222–177.76	1/40	[30]
59	Spain	3.8	2.7	63.6	62.0	20.0	18.0	1348.11	2.13	A	312.88	B	2.222–177.76	1/40	[30]
60	Spain	5.0	1.0	123.0	48.0	27.0	25.0	4309.14	2.13	a	NA	–	2.222–177.76	1/40	[30]
61	Spain	5.7	2.3	128.6	65.0	14.0	21.0	2267.76	2.94	A	351.74	B	2.222–177.76	1/40	[30]
62	China	2.8	0.5	52.2	63.0	18.0	19.0	2448.27	2.78	A	507.23	B	2.222–177.76	1/40	[30]
63	Spain	5.0	3.6	86.2	48.0	31.0	21.0	2356.12	2.56	a	566.23	B	2.222–177.76	1/40	[30]
64	China	8.6	1.2	216.1	49.0	29.0	22.0	2940.36	2.70	A	872.31	b	2.222–177.76	1/40	[30]
65	Spain	4.5	3.0	67.6	59.0	21.0	20.0	2152.16	2.50	A	468.18	B	2.222–177.76	1/40	[30]
66	China	7.6	0.9	52.6	61.0	20.0	19.0	2536.59	2.70	A	613.10	B	2.222–177.76	1/40	[30]
67	Spain	4.0	10.9	116.4	55.0	22.0	23.0	2074.13	2.22	A	569.98	B	2.222–177.76	1/40	[30]
68	Spain	3.9	7.3	215.0	49.0	19.0	32.0	6928.09	1.54	a	NA	–	2.222–177.76	1/40	[30]
69	Spain	4.1	1.2	44.7	43.0	39.0	18.0	1157.25	1.43	A	580.29	B	2.222–177.76	1/40	[30]
70	Spain	5.1	3.0	98.5	22.0	62.0	16.0	997.20	2.04	a	162.85	b	2.222–177.76	1/40	[30]
71	Spain	4.3	1.8	49.5	39.0	47.0	14.0	1787.98	2.22	A	374.43	B	2.222–177.76	1/40	[30]

72	Spain	3.7	5.0	76.9	35.0	45.0	20.0	1643.77	1.85	A	521.45	B	2.222–177.76	1/40	[30]
73	Spain	4.5	1.3	43.2	26.0	51.0	23.0	1741.41	1.75	A	665.04	B	2.222–177.76	1/40	[30]
74	Spain	7.9	2.6	138.0	36.0	45.0	19.0	3504.97	1.67	A	NA	–	2.222–177.76	1/40	[30]
75	Spain	4.5	5.3	116.5	30.0	55.0	15.0	1631.31	2.00	a	416.44	B	2.222–177.76	1/40	[30]
76	Spain	6.4	1.8	125.0	26.0	58.0	16.0	712.18	1.41	A	313.01	B	2.222–177.76	1/40	[30]
77	China	7.1	0.8	239.5	41.0	42.0	17.0	2591.79	2.70	A	584.82	B	2.222–177.76	1/40	[30]
78	Spain	4.2	2.4	42.5	27.0	56.0	17.0	1151.66	1.72	A	358.10	b	2.222–177.76	1/40	[30]
79	Spain	4.2	2.5	46.5	25.0	60.0	15.0	1183.12	1.43	a	599.33	B	2.222–177.76	1/40	[30]
80	Spain	8.0	1.0	121.0	24.0	59.0	17.0	3442.68	3.70	A	682.13	B	2.222–177.76	1/40	[30]
81	Spain	5.0	3.1	129.3	30.0	47.0	23.0	957.37	1.69	A	277.48	B	2.222–177.76	1/40	[30]
82	China	6.8	0.6	71.2	40.0	42.0	18.0	3255.03	1.11	A	NA	–	2.222–177.76	1/40	[30]
83	Spain	6.2	5.1	161.0	25.1	65.8	9.1	NA	NA	–	419.38	B	2.5–25	1/250	[133]
84	Spain	4.0	10.9	116.0	NA	NA	NA	642.00	0.43	a	NA	–	25–200	1/625	[23]
85	Spain	6.4	4.6	303.1	NA	NA	NA	2080.00	0.39	a	NA	–	25–200	1/625	[23]
86	China	6.8	0.6	71.2	25.8	27.4	46.8	11.03	0.89	A	NA	–	5–30	1/25	[113]
87	China	8.5	0.7	61.0	33.3	52.2	14.5	26.98	1.10	A	NA	–	5–30	1/25	[113]
88	China	6.5	1.4	163.2	73.2	18.6	8.2	6.15	0.82	A	10.06	B	5–30	1/25	[113]
89	China	5.7	1.3	185.6	NA	NA	18.0	49.91	1.09	a	NA	–	2.0–80.0	1/50	[114]
90	China	6.9	0.7	352.0	22.0	51.0	27.0	NA	NA	–	1735.00	b	0.01–0.04	NA	[74]
91	China	4.6	1.6	600.0	33.0	38.0	29.0	NA	NA	–	1481.00	b	0.01–0.04	NA	[74]
92	India	7.5	2.9	265.0	25.0	55.0	20.0	NA	NA	–	1136.00	b	0.01–0.04	NA	[74]
93	China	8.3	0.5	84.2	17.0	65.0	18.0	NA	NA	–	1359.00	b	0.01–0.04	NA	[74]
94	China	6.3	1.1	151.2	70.4	26.2	3.4	89.09	0.68	A	386.74	B	13.33–88.89 [6]	1/200	[114]
95	China	9.0	0.3	210.5	NA	NA	21.0	3102.00	1.35	a	NA	–	0.2–29	1/58.33	[11]
96	Spain	5.9	2.0	150.0	13.6	55.5	30.9	664.00	1.09	A	696.20	B	0.5–10	1/50	[6]
97	Spain	4.3	3.4	59.0	29.1	41.8	29.1	399.00	1.14	A	403.17	B	0.5–10	1/50	[6]

98	China	6.2	2.2	198.8	7.7	58.3	34.0	255.00	1.20	A	239.54	b	0.5–10	1/50	[6]
99	China	5.5	0.6	136.9	51.5	28.9	19.6	367.00	1.22	A	366.41	B	0.5–10	1/50	[6]
100	UK	6.0	4.1	251.1	52.5	33.8	13.7	NA	NA	–	499.72	B	0.5–10	1/50	[6]
101	UK	5.3	3.2	142.1	36.7	52.0	11.3	NA	NA	–	718.63	b	0.5–10	1/50	[6]
102	UK	6.7	3.5	242.8	56.4	32.7	10.9	NA	NA	–	254.19	B	0.5–10	1/50	[6]
103	China	5.5	0.6	136.9	36.7	42.8	20.5	828.00	1.41	A	1100.20	B	0.5–10	1/50	[6]
104	Spain	4.5	1.1	42.5	81.0	10.3	8.7	1601.00	1.28	a	NA	–	0.5–10	1/50	[6]
105	China	8.1	0.1	35.1	38.6	37.7	23.7	240.00	1.28	A	217.05	B	0.5–10	1/50	[6]
106	China	6.9	1.2	215.0	76.0	13.5	10.5	295.00	1.56	a	262.10	B	0.5–10	1/50	[6]
107	UK	4.8	1.3	44.2	72.2	17.5	10.3	NA	NA	A	396.63	B	0.5–10	1/50	[6]
108	China	6.2	2.2	198.8	47.6	30.2	22.2	322.00	1.41	A	304.87	B	0.5–10	1/50	[6]
109	Spain	4.0	1.7	52.1	14.9	36.2	48.9	1095.00	0.98	A	1072.30	B	1.0–10.0	1/50	[134]
110	Spain	4.4	2.4	58.9	14.9	36.2	48.9	1093.96	0.98	A	1071.30	B	1–10.0	1/50	[135]
111	Spain	4.4	2.1	52.9	29.2	45.2	25.6	1475.71	1.46	A	NA	–	1–10.0	1/50	[135]
112	China	9.0	0.3	210.5	25.8	27.4	46.8	11.03	0.89	A	NA	–	NA	NA	[123]
113	China	7.1	1.2	154.2	73.2	18.6	8.2	6.15	0.82	A	10.06	B	NA	NA	[123]
114	China	6.2	2.2	19.9	18.7	53.9	27.4	0.28	1.31	A	NA	–	5–110	1/20	[136]
115	Spain	8.2	1.5	106.0	28.1	38.3	33.6	NA	NA	–	1794.73	b	5.0–200.0	1/50	[125]
116	Spain	7.2	2.5	110.0	20.2	42.3	37.5	NA	NA	–	1940.89	b	5.0–200.0	1/50	[125]
117	Spain	3.8	2.0	47.4	68.9	25.7	5.4	1106.00	0.83	A	NA	–	5–150	1/250	[137]
118	Spain	4.4	3.7	51.3	82.3	16.5	1.2	1127.00	0.80	A	NA	–	5–150	1/250	[137]
119	China	6.2	2.2	198.8	50.4	38.2	11.4	778.00	0.80	A	NA	–	5–150	1/250	[137]

[1] Sub-datasets "A" and "a" were used for development and validation of the models for estimating K_f, respectively.

[2] Sub-datasets "B" and "b" were used for development and validation of the models for estimating K_d, respectively.

[3] $CaCl_2$ was used as background electrolyte in all batch sorption tests, unless otherwise stated.

[4] "NA" means that the value was not reported in the cited reference.

[5] "–" denotes an exclusion from any sub-datasets.

[6] NaCl was used as background electrolyte in batch sorption test.

Table S8. CTC sorption parameters and associated soil properties.

No.	Origin	Soil properties						Freundlich			Linear		Initial antibiotic concentration range [3]	Solid/Liquid ratio	Reference
		pH	OC	CEC	Sand	Silt	Clay	K_f	*n*	Sub-dataset [1]	K_d	Sub-dataset [2]			
			%	mmol kg^{-1}	%	%	%	mg$^{1-1/n}$ L$^{1/n}$ kg^{-1}			L kg^{-1}		mg L^{-1}	g mL^{-1}	
1	China	7.1	1.2	154.2	13.4	39.5	47.1	NA [4]	NA	– [5]	3854.78	b	1–10. 0	1/50	[106]
2	China	6.5	1.4	163.2	17.6	37.1	45.3	NA	NA	–	4168.69	b	1–10. 0	1/50	[106]
3	Spain	5.0	3.1	129.3	58.0	22.0	20.0	3019.69	1.96	A	1365.20	B	2.395–191.6	1/40	[13]
4	Spain	5.1	3.0	98.5	64.0	17.0	19.0	2115.77	2.08	A	608.09	B	2.395–191.6	1/40	[13]
5	Spain	4.3	3.4	59.2	58.0	19.0	22.0	4535.37	3.03	A	1727.40	B	2.395–191.6	1/40	[13]
6	Spain	4.5	2.8	53.1	61.0	18.0	20.0	3926.52	2.38	a	1722.50	B	2.395–191.6	1/40	[13]
7	Spain	4.4	2.4	58.9	62.0	20.0	17.0	5608.20	3.13	a	3115.60	B	2.395–191.6	1/40	[13]
8	Spain	4.0	1.7	52.1	53.0	27.0	20.0	3332.58	2.56	a	1028.80	B	2.395–191.6	1/40	[13]
9	Spain	3.8	2.0	47.4	60.0	19.0	21.0	3826.57	2.17	A	1863.20	B	2.395–191.6	1/40	[13]
10	Spain	4.7	3.9	65.9	51.0	21.0	29.0	5601.31	2.44	A	3860.70	B	2.395–191.6	1/40	[13]
11	Spain	4.9	3.8	64.8	65.0	17.0	19.0	5217.18	4.00	A	1814.90	B	2.395–191.6	1/40	[13]
12	Spain	4.4	3.7	51.3	62.0	20.0	19.0	3381.60	2.33	A	1277.60	B	2.395–191.6	1/40	[13]
13	Spain	4.2	2.4	42.5	65.0	15.0	21.0	2558.73	2.00	A	959.22	B	2.395–191.6	1/40	[13]
14	Spain	4.1	1.2	44.7	64.0	19.0	17.0	2046.74	2.50	a	359.73	B	2.395–191.6	1/40	[13]
15	Spain	4.2	2.5	46.5	66.0	16.0	18.0	3077.65	2.17	a	1179.50	B	2.395–191.6	1/40	[13]
16	Spain	3.9	1.7	52.1	43.0	27.0	30.0	4369.41	2.86	a	1702.00	B	2.395–191.6	1/40	[13]
17	Spain	4.4	1.3	38.8	66.0	15.0	18.0	3232.53	3.23	A	582.47	B	2.395–191.6	1/40	[13]
18	Spain	4.1	1.3	40.0	68.0	14.0	17.0	2945.58	3.23	A	451.88	B	2.395–191.6	1/40	[13]
19	Spain	3.8	2.7	63.6	54.0	24.0	21.0	1432.91	1.43	A	757.63	B	2.395–191.6	1/40	[13]

20	Spain	3.7	1.7	44.4	59.0	22.0	20.0	3256.52	3.45	A	510.36	B	2.395–191.6	1/40	[13]
21	Spain	4.3	1.1	40.8	70.0	12.0	18.0	2109.71	2.94	A	251.08	B	2.395–191.6	1/40	[13]
22	Spain	4.0	1.6	47.4	55.0	22.0	23.0	3131.56	3.03	A	616.02	B	2.395–191.6	1/40	[13]
23	Spain	4.0	1.9	46.1	58.0	23.0	19.0	3733.48	3.03	A	994.64	B	2.395–191.6	1/40	[13]
24	Spain	4.4	2.1	52.9	61.0	16.0	23.0	3980.49	2.56	A	1584.60	B	2.395–191.6	1/40	[13]
25	Spain	4.1	2.1	52.5	55.0	26.0	19.0	3293.55	2.94	A	752.46	B	2.395–191.6	1/40	[13]
26	Spain	3.8	1.6	41.5	57.0	25.0	18.0	3298.54	3.03	a	708.03	B	2.395–191.6	1/40	[13]
27	Spain	4.0	2.0	43.0	64.0	19.0	17.0	2146.67	3.70	a	147.08	B	2.395–191.6	1/40	[13]
28	Spain	4.2	1.1	42.4	69.0	13.0	18.0	2700.61	3.33	A	331.70	B	2.395–191.6	1/40	[13]
29	Spain	4.5	1.1	42.5	67.0	14.0	19.0	2834.59	3.33	A	367.73	B	2.395–191.6	1/40	[13]
30	Spain	4.5	5.3	116.5	41.0	26.0	34.0	5368.53	1.72	a	4473.20	B	2.395–191.6	1/40	[13]
31	Spain	4.2	3.3	65.1	57.0	20.0	23.0	4189.42	2.94	A	1447.20	B	2.395–191.6	1/40	[13]
32	Spain	6.2	1.7	164.7	56.0	20.0	24.0	1098.94	1.39	A	561.44	B	2.395–191.6	1/40	[13]
33	Spain	3.7	5.0	76.9	51.0	22.0	27.0	5414.23	3.13	a	2800.50	B	2.395–191.6	1/40	[13]
34	Spain	4.1	1.8	44.9	48.0	23.0	29.0	4047.42	3.23	A	1113.40	B	2.395–191.6	1/40	[13]
35	Spain	4.5	1.3	43.2	63.0	17.0	19.0	2771.62	2.94	A	481.73	B	2.395–191.6	1/40	[13]
36	Spain	4.7	2.6	71.8	53.0	22.0	26.0	4310.50	2.22	A	2334.50	B	2.395–191.6	1/40	[13]
37	Spain	4.2	1.4	56.2	58.0	22.0	20.0	5572.27	2.70	A	NA	–	2.395–191.6	1/40	[13]
38	Spain	5.0	2.4	61.9	66.0	17.0	18.0	4315.45	2.56	A	1934.00	B	2.395–191.6	1/40	[13]
39	Spain	4.3	1.8	49.5	62.0	22.0	16.0	4085.49	2.50	A	1756.00	B	2.395–191.6	1/40	[13]
40	Spain	4.3	2.5	63.9	59.0	19.0	22.0	5572.21	3.13	A	3055.40	B	2.395–191.6	1/40	[13]
41	Spain	4.2	2.2	54.5	63.0	20.0	17.0	5153.27	3.13	A	2412.70	B	2.395–191.6	1/40	[13]
42	Spain	4.2	2.3	43.9	65.0	18.0	17.0	2089.82	1.72	a	904.62	B	2.395–191.6	1/40	[13]
43	Spain	4.4	2.5	54.0	62.0	20.0	18.0	2319.78	1.82	a	970.95	B	2.395–191.6	1/40	[13]
44	Spain	4.6	3.8	68.2	48.0	27.0	25.0	7169.34	1.79	A	NA	–	2.395–191.6	1/40	[13]
45	Spain	4.1	3.1	53.3	65.0	14.0	21.0	4463.43	2.56	A	2102.50	B	2.395–191.6	1/40	[13]

46	Spain	4.5	3.7	67.3	63.0	18.0	19.0	5868.17	3.13	A	3575.80	B	2.395–191.6	1/40	[13]
47	Spain	4.1	2.7	50.3	48.0	31.0	21.0	5910.19	2.94	A	3838.00	b	2.395–191.6	1/40	[13]
48	Spain	4.2	3.3	54.5	49.0	29.0	22.0	6204.15	2.94	A	4414.10	b	2.395–191.6	1/40	[13]
49	Spain	4.5	3.5	48.2	59.0	21.0	20.0	6068.14	3.13	A	3960.50	B	2.395–191.6	1/40	[13]
50	Spain	4.5	3.7	60.4	61.0	20.0	19.0	6194.12	3.13	A	4217.10	b	2.395–191.6	1/40	[13]
51	Spain	4.4	2.8	87.8	55.0	22.0	23.0	4627.40	2.63	A	2224.40	B	2.395–191.6	1/40	[13]
52	Spain	6.4	4.6	303.1	44.0	39.0	18.0	4878.50	1.96	a	3430.90	B	2.395–191.6	1/40	[13]
53	Spain	5.9	2.0	149.6	22.0	62.0	16.0	3041.59	2.86	A	652.43	B	2.395–191.6	1/40	[13]
54	Spain	4.5	3.0	67.6	39.0	47.0	14.0	4242.48	2.44	A	1991.50	B	2.395–191.6	1/40	[13]
55	Spain	5.5	2.8	110.9	35.0	45.0	20.0	NA	NA	–	475.34	B	2.395–191.6	1/40	[13]
56	Spain	6.2	2.9	234.1	26.0	51.0	23.0	5171.34	2.56	A	3035.50	B	2.395–191.6	1/40	[13]
57	Spain	5.1	4.6	167.7	37.0	45.0	19.0	8176.99	2.44	A	NA	–	2.395–191.6	1/40	[13]
58	Spain	5.2	1.5	67.5	30.0	55.0	15.0	5408.25	2.94	A	2975.50	B	2.395–191.6	1/40	[13]
59	Spain	5.7	2.3	128.6	26.0	58.0	16.0	2826.69	2.13	A	1031.80	B	2.395–191.6	1/40	[13]
60	Spain	4.3	4.3	60.4	41.0	42.0	17.0	7505.87	3.57	A	NA	–	2.395–191.6	1/40	[13]
61	Spain	6.4	1.7	125.4	27.0	56.0	17.0	4138.51	2.33	A	2009.50	B	2.395–191.6	1/40	[13]
62	Spain	5.4	2.0	144.5	25.0	60.0	15.0	4340.45	2.56	A	1961.80	B	2.395–191.6	1/40	[13]
63	Spain	5.0	3.6	86.2	25.0	59.0	17.0	8052.94	2.70	A	NA	–	2.395–191.6	1/40	[13]
64	Spain	5.5	1.6	80.3	30.0	47.0	23.0	3604.55	2.50	A	1301.10	B	2.395–191.6	1/40	[13]
65	Spain	4.3	1.1	41.0	64.7	14.0	21.3	642.72	2.78	A	NA	–	23.02–276.24	1/40	[109]
66	Spain	5.9	2.0	150.0	29.3	49.3	21.4	541.83	2.08	A	NA	–	23.02–276.24	1/40	[109]
67	Spain	6.4	1.8	125.0	27.3	51.3	21.4	977.25	1.69	A	224.50	B	23.02–276.24	1/40	[109]
68	China	7.2	1.2	187.0	40.2	12.5	47.3	887.00	1.56	A	NA	–	50	1/100	[37]
69	China	7.6	0.9	133.0	41.1	22.5	37.4	870.00	1.16	A	625.00	b	50–100	1/25	[139]
70	China	6.9	0.7	352.0	NA	NA	NA	847.00	0.43	a	NA	–	25–200	1/62.5	[23]
71	China	4.6	1.6	600.0	NA	NA	NA	1635.00	0.57	a	NA	–	25–200	1/62.5	[23]

72	China	6.8	1.6	178.0	7.3	59.2	33.5	870.96	0.82	A	476.43	b	5–20	1/25	[112]
73	China	7.1	2.7	282.0	6.1	65.3	28.6	1499.68	0.75	A	746.45	B	5–20	1/25	[112]
74	China	8.3	0.5	84.2	NA	NA	18.0	302.00	1.01	a	300.00	b	2.0–80.0	1/50	[114]
75	China	8.0	0.6	104.4	62.4	24.2	13.4	1135.01	1.69	A	933.25	B	10–30	1/100	[140]
76	China	7.6	0.3	178.9	36.3	41.6	22.1	1250.26	0.99	A	1258.93	B	10–50	1/100	[140]
77	China	6.6	1.5	175.0	38.0	46.5	15.5	2618.18	1.32	A	2884.03	B	20–80	1/100	[140]
78	China	8.0	0.6	104.4	62.4	24.2	13.4	1148.15	1.69	A	NA	–	10–30	1/100	[140]
79	China	7.6	0.3	178.9	36.3	41.6	22.1	NA	NA	–	447.30	B	10–50	1/100	[140]
80	China	6.6	1.5	175.0	38.0	46.5	15.5	2630.27	1.33	A	NA	–	20–80	1/100	[140]
81	China	5.8	0.5	240.8	34.0	37.8	28.2	1258.93	0.99	A	1055.60	B	30–80	1/100	[140]
82	China	7.2	0.6	NA	NA	NA	NA	950.60	1.56	a	NA	–	10–50	1/100	[142]
83	China	7.2	2.4	NA	NA	NA	NA	1032.76	1.15	a	NA	–	10–50	1/100	[142]
84	China	7.2	0.7	NA	NA	NA	NA	916.22	1.38	a	NA	–	10–50	1/100	[142]
85	China	6.5	0.8	NA	NA	NA	NA	922.57	1.59	a	NA	–	10–50	1/100	[142]
86	China	8.7	0.6	133.0	16.6	73.8	9.6	2054.00	1.14	A	348.00	B	10–80	1/100	[143]
87	USA	7.5	2.9	265.0	NA	NA	21.0	NA	NA	–	2093.90	b	0.2–29	1/58.33	[11]
88	Spain	8.2	1.1	171.0	13.6	55.5	30.9	1745.00	0.90	A	1502.10	B	0.5–10	1/50	[6]
89	Spain	8.1	2.3	154.0	29.1	41.8	29.1	1002.00	0.98	A	983.39	B	0.5–10	1/50	[6]
90	Spain	8.0	1.0	121.0	7.7	58.3	34.0	417.00	1.35	A	429.80	b	0.5–10	1/50	[6]
91	Spain	7.9	2.6	138.0	51.5	28.9	19.6	NA	NA	–	603.87	B	0.5–10	1/50	[6]
92	Spain	6.7	5.4	220.0	52.5	33.8	13.7	NA	NA	–	960.66	b	0.5–10	1/50	[6]
93	Spain	6.5	8.8	243.0	36.7	52.0	11.3	NA	NA	–	1471.00	b	0.5–10	1/50	[6]
94	Spain	5.3	1.9	65.0	56.4	32.7	10.9	323.00	1.72	A	290.20	B	0.5–10	1/50	[6]
95	Spain	5.0	1.0	123.0	36.7	42.8	20.5	870.00	1.32	A	1104.50	B	0.5–10	1/50	[6]
96	Spain	3.9	7.3	215.0	81.0	10.3	8.7	NA	NA	–	3422.60	b	0.5–10	1/50	[6]
97	Spain	8.2	1.5	106.0	38.6	37.7	23.7	511.00	1.16	A	535.81	B	0.5–10	1/50	[6]

98	Spain	7.2	2.5	110.0	76.0	13.5	10.5	NA	NA	–	366.37	B	0.5–10	1/50	[6]
99	Spain	6.0	1.7	121.0	47.6	30.2	22.2	445.00	1.37	A	468.62	B	0.5–10	1/50	[6]
100	China	6.2	2.2	198.8	14.9	36.2	48.9	4877.00	0.75	A	2559.30	B	1–10. 0	1/50	[144]
101	China	7.5	1.4	180.0	29.1	21.3	49.6	3234.40	1.27	A	2036.90	b	60–240	1/100	[145]
102	China	7.2	1.6	242.7	28.9	27.4	43.7	2578.10	1.58	A	870.03	b	60–240	1/100	[145]
103	China	9.4	0.9	136.0	29.7	32.1	38.2	3566.20	1.26	A	2337.50	B	60–240	1/100	[145]
104	China	5.0	8.7	NA	14.8	78.2	0.1	NA	NA	–	3073.00	b	10.0–50.0	1/100	[138]

[1] Sub-datasets “A” and “a” were used for development and validation of the models for estimating K_f, respectively.

[2] Sub-datasets “B” and “b” were used for development and validation of the models for estimating K_d, respectively.

[3] $CaCl_2$ was used as background electrolyte in all batch sorption tests.

[4] “NA” means that the value was not reported in the cited reference.

[5] “–” denotes an exclusion from any sub-datasets.

Table S9. pH distribution of the soils in sub-datasets "A" (for K_f) and "B" (for K_d) and main antibiotic species in soil water.

Antibiotic	pH	Main species	K_f		K_d	
			N [1]	Percentage (%)	N	Percentage (%)
SCP	<pK_{a1}	SCP^+	0	0	0	0
	pK_{a1}–pK_{a2}	SCP^0	49	72.1	58	72.5
	>pK_{a2}	SCP^-	19	27.9	22	27.5
SMT	<pK_{a1}	SMT^+	0	0	0	0
	pK_{a1}–pK_{a2}	SMT^0	101	94.4	108	94.7
	>pK_{a2}	SMT^-	6	5.6	6	5.3
SDZ	<pK_{a1}	SDZ^+	0	0	0	0
	pK_{a1}–pK_{a2}	SDZ^0	46	86.8	68	81.9
	>pK_{a2}	SDZ^-	7	13.2	15	18.1
SMX	<pK_{a1}	SMX^+	0	0	0	0
	pK_{a1}–pK_{a2}	SMX^0	20	40.8	25	43.9
	>pK_{a2}	SMX^-	29	59.2	32	56.1
OTC	<pK_{a1}	OTC^+	0	0	0	0
	pK_{a1}–pK_{a2}	OTC^0	86	82.7	82	87.2
	>pK_{a2}	OTC^-, OTC^{2-}	18	17.3	12	12.8
TC	<pK_{a1}	TC^+	0	0	0	0
	pK_{a1}–pK_{a2}	TC^0	72	85.7	62	92.5
	>pK_{a2}	TC^-, TC^{2-}	12	14.3	5	7.5
CTC	<pK_{a1}	CTC^+	0	0	0	0
	pK_{a1}–pK_{a2}	CTC^0	62	84.9	63	87.5
	>pK_{a2}	CTC^-, CTC^{2-}	10	15.1	9	12.5

[1] Number of soils.

Table S10. SDM sorption parameters, associated soil properties, and the performance of the model established for SMT in this study.

No.	Origin	Soil properties						Freundlich		Initial antibiotic concentration range [1]	Solid/Liquid ratio	RMSE (RMSE/SD)	NSE	Reference
		pH	OC	CEC	Sand	Silt	Clay	K_f	n					
			%	mmol kg⁻¹	%	%	%	$mg^{1-1/n}\ L^{1/n}\ kg^{-1}$		mg L⁻¹	g mL⁻¹			
1	Brazil	5.0	0.9	19.3	91.1	1.8	6.2	1.40	1.15	5–100	1/2	2.23 (34.1%)	0.88	[53]
2	Brazil	4.9	1.7	52.7	14.9	30.2	54.6	4.60	1.32	5–100	1/2			[53]
3	Brazil	4.1	1.4	51.9	52.9	10.5	36.2	4.60	1.33	5–100	1/2			[53]
4	Brazil	4.4	1.9	66.0	43.5	7.0	49.2	6.40	1.39	5–100	1/2			[53]
5	China	4	1.0	96.9	16.9	67.2	15.9	2.02	1.05	0.05–1	1/2.5			[84]
6	China	5.8	6.1	312.0	11.3	73.1	15.6	4.32	1.01	0.05–1	1/2.5			[84]
7	China	6.2	5.0	180.0	4.8	75.8	19.4	3.50	1.02	0.05–1	1/2.5			[84]
8	China	7.6	2.4	106.0	16.5	74.3	9.2	0.37	1.64	0.05–1	1/2.5			[84]
9	China	5.6	1.0	218.0	2.4	82.9	14.7	0.10	1.19	0.05–1	1/2.5			[84]
10	Poland	5.8	24.5	270.0	NA [2]	NA	94.0	24.95	1.39	0.625–80	1/25			[75]
11	Poland	7.4	0.1	30.0	NA	NA	0.2	0.07	1.49	0.625–80	1/2			[75]
12	China	7.0	1.5	1250.0	81.2	17.0	1.8	0.22	1.01	0.1–10	1/2.5			[146]

[1] $CaCl_2$ was used as background electrolyte in all batch sorption tests.

[2] "NA" means that the value was not reported in the cited reference.
